# Therapeutic potential of flavonoids from traditional Chinese medicine in pancreatic cancer treatment

**DOI:** 10.3389/fnut.2024.1477140

**Published:** 2024-11-22

**Authors:** Qi Wan, Qing Ren, Shuangying Qiao, Aiping Lyu, Xingwei He, Fangfei Li

**Affiliations:** ^1^Acupuncture Department, Affiliated Hospital of Jiangxi University of Chinese Medicine, Nanchang, China; ^2^Graduate School, Jiangxi University of Chinese Medicine, Nanchang, China; ^3^Institute of Integrated Bioinfomedicine and Translational Science, School of Chinese Medicine, Hong Kong Baptist University, Kowloon Tong, Hong Kong SAR, China; ^4^Li Ka Shing Faculty of Medicine, The University of Hong Kong, Pokfulam, Hong Kong SAR, China

**Keywords:** flavonoids, traditional Chinese herbal medicine, treatment modalities, pancreatic cancer, anti-cancer efficacy

## Abstract

Pancreatic cancer (PC) is a highly aggressive malignancy with rising mortality rates globally. Its diagnosis is often challenging due to its asymptomatic nature in the early stages. Consequently, most patients receive a poor prognosis, with low survival rates within 5 years, as the disease is typically detected at an advanced stage, complicating effective treatment. Flavonoids, especially those derived from traditional Chinese herbal medicines, have attracted considerable attention for their potent anti-PC properties. This review highlights the therapeutic potential of these bioactive compounds, which modulate key biological pathways, making them promising candidates for PC intervention. Their mechanisms of action include the regulation of autophagy, apoptosis, cell growth, epithelial-mesenchymal transition, and oxidative stress, as well as enhancing chemotherapeutic sensitivity, exerting antiangiogenic effects, and potentially boosting immunomodulatory responses. The demonstrated benefits of these natural compounds in cancer management have spurred extensive academic interest. Beyond their role as anti-cancer agents, flavonoids may provide both preventive and therapeutic advantages for PC, resonating with the core principles of traditional Chinese medicine for disease prevention and holistic treatment.

## 1 Introduction

Pancreatic cancer (PC), renowned for its aggressiveness, is a significant cause of cancer-related death in developed countries and is among the deadliest cancers worldwide. Predictions indicate that by 2030, the incidence of PC will increase to second place in terms of cancer-related mortality in the United States. This projection accounts for nearly 7–8% of the aggregate deaths attributed to cancer (1). The pancreas bears the brunt of this highly formidable cancer, known for its propensity to vigorously advance. Regrettably, the prognosis for those afflicted with PC is typically grim. The disease’s lethal nature stems from its proclivity for localized growth along perineural and vascular pathways, accompanied by early instances of distant metastasis. These characteristics collectively hinder the feasibility of curative surgical intervention in the majority of patients (2). As a result, its survival rate is low, and the available treatment options are limited (3).

Advances in the treatment of PC are urgently needed. Naturally occurring compounds, especially flavonoids, are considered important sources for the development of novel anti-cancer drugs and have shown great potential in this regard (4).

Flavonoid-rich herbs are a class of bioactive compounds that have been extensively studied and incorporated into traditional Chinese medicine (TCM) for centuries (5). Abundant reservoirs of these natural polyphenolic compounds can be traced within a diverse array of fruits, vegetables, and herbs extensively employed within formulations of traditional Chinese medicine (TCM). Among these compounds, flavonoids are known for their multifaceted pharmacological properties, including anti-inflammatory effects. Notably, these pharmacological properties are in harmony with the fundamental principles of traditional Chinese medicine, which focus on restoring balance and calm.

In traditional Chinese medicine, flavonoid-containing herbs have been used for a variety of medicinal purposes, including treatment or prevention of cardiovascular disease, cancer and inflammation. This ancient practice reflects a deep-rooted understanding of using flavonoid-rich botanicals alongside other natural compounds to create synergistic healing effects. Conflicting evidence on flavonoid’s dual pro-oxidant and anti-carcinogenic properties contributes to this complexity, leaving their full impact open to further exploration.

Thus, we have undertaken to review the potential of flavonoids from various sources in TCM for their capacity against PC. In this review, the literature covered in the last 20 years. The current study synthesized relevant insights on PC from popular databases such as PubMed, Science Direct Web of Science and Europe PMC. We searched using terms “PC” and limited the result to pharmacology and flavonoids, Chinese medicine in combination with MeSH Terms or all fields as appropriate. Our primary goal is to give a thorough reference for future efforts in novel drug development towards cancer chemoprevention, and therapy approaches against PC.

## 2 Flavonoid compounds and their characteristics

### 2.1 Definition and classification of flavonoid compounds

Flavonoids, which are abundant in plants, represent a category of innate polyphenolic compounds distinguished by their distinct chemical configurations. Their hallmark is the arrangement of 15 carbon atoms intricately linked to form three interlocking rings, resulting in the foundational structure termed C6-C3-C6. These three rings comprise two benzene rings (C6) tethered together through a heterocyclic pyran ring (C3).

This distinct arrangement contributes to the diverse subgroups of flavonoids, each with specific variations in hydroxyl and methoxy groups at different positions on the rings (6). The various subgroups include flavones, flavonols, flavanones, flavanonols, flavan-3-ols (catechins), and Anthocyanins (7–9). The specific arrangement and presence of different functional groups in their chemical structure play pivotal roles in determining the biological activities and health benefits of flavonoids.

### 2.2 Natural sources of flavonoid compounds

Flavonoid compounds are abundantly found in nature, predominantly in various plants and fruits. Apples, for example, encompass a range of flavonoids, including Quercetin, catechin, and epicatechin. Similarly, berries such as strawberries, blueberries, raspberries, and blackberries are rich in Anthocyanins. Citrus fruits, including oranges, lemons, limes, and grapefruits, are notable for their high flavanone content, particularly hesperidin and Naringenin. Additionally, grapes and cherries, as well as vegetables such as *onions*, *broccoli*, *kale*, and *tomatoes*, are significant sources of flavonoids. In addition, tea, including g*reen tea*, *black tea*, and *white tea*, is a notable source of flavonoids such as catechins and flavonols (10, 11). Cocoa, dark chocolate, legumes such as soybeans and lentils, and nuts such as almond, walnut, and pecans also contribute to flavonoid intake.

Chinese medicinal herbs are rich in flavonoids, which offer a wide range of health benefits. *Ginkgo biloba*, for example, enhances blood circulation and cognitive function in combination with flavonoids such as Quercetin and Kaempferol. *Green tea* is known for its antioxidant catechins, which aid in mental alertness and cardiovascular health. *Honeysuckle* and *Sophora japonica* contain anti-inflammatory flavonoids such as Quercetin and Rutin, whereas *Hawthorn* supports cardiovascular health via similar compounds (12). *Licorice* root Isoliquiritigenin and Chrysanthemum Apigenin have anti-inflammatory and antioxidant effects. *Scutellaria* baicalensis stands out for its antibacterial and anti-cancer properties, highlighting the diverse and beneficial effects of flavonoids in traditional Chinese medicine.

A broad spectrum of biological activities is shown by flavonoid compounds, coupled with distinctive attributes that render them compelling subjects for exploration within scientific research and medical contexts. Renowned for their robust antioxidant attributes, these innate polyphenolic compounds play pivotal roles in counteracting free radicals and safeguarding cells against the perils of oxidative stress and the resulting harm.

The potential of flavonoid compounds to exert anti-cancer effects has been a topic of significant discussion and research in the scientific community. Flavonoids, as natural polyphenolic compounds, have shown promising results in inhibiting tumor growth, inducing cancer cell apoptosis, and in conjunction with diverse pathways of cellular signaling implicated in the progression of cancer. Their formidable antioxidant characteristics assume a pivotal role in nullifying free radicals and safeguarding cells against oxidative harm, which is closely linked to cancer development. Additionally, the anti-inflammatory effects of flavonoids help modulate inflammatory pathways, reducing chronic inflammation, which contributes to cancer initiation and promotion (13).

Studies have highlighted the potential of specific flavonoids, including but not limited to Quercetin and Kaempferol, to target specific cancer cell types and exhibit selective cytotoxicity toward cancer cells while sparing healthy cells. These compounds have the ability to impede the proliferation of cancer cells, trigger cell cycle arrest, and facilitate apoptosis, making them promising candidates for future cancer therapies.

Furthermore, flavonoids could increase the responsiveness of cancer cells to established chemotherapy and radiotherapy, thereby increasing the effectiveness of these therapeutic interventions (14). This combined approach has the potential to reduce treatment resistance and improve overall treatment outcomes.

#### 2.2.1 Epigallocatechin-3-gallate

**Figure 1:**
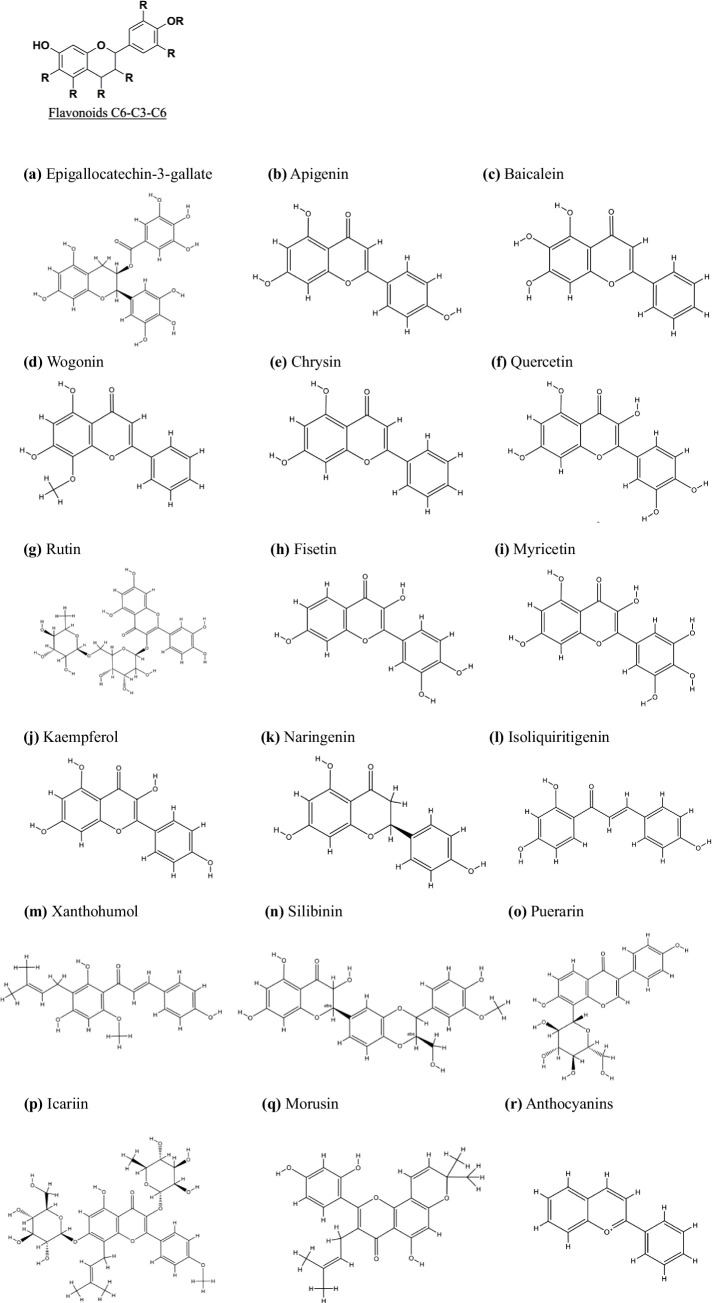
Chemical structures and classification of flavonoid (C6-C3-C6) subgroups for PC treatment. The flavonoid (C6-C3-C6) subclasses include Flavanols **(A)** Epigallocatechin-3-gallate, Flavones **(B)**: Apigenin, **(C)**: Baicalein, **(D)**: Wogonin, **(E)**: Chrysin, Flavonols **(F)**: Quercetin, **(G)**: Rutin, **(H)**: Fisetin, **(I)**: Myricetin, **(J)**: Kaempferol, **(P)**: Icariin, Flavanones **(K)**: Naringenin, **(Q)**: Morusin, Flavanonols **(N)**: Silibinin, Chalcones **(M)**: Xanthohumol, Isoflavones **(L)**: Isoliquiritigenin, **(O)**: Puerarin, and Anthocyanidins **(R)**: Anthocyanins. All chemical structures were drawn using ChemDraw software version 8.0.

Epigallocatechin-3-gallate (EGCG), a compound isolated from *green tea* (Figure 1A), exhibits significant anti-cancer activity (15, 16). Hypoxia-inducible factor-1 (*HIF-1*), a heterodimeric transcription factor, plays a critical role in the progression of cancer, contributing to its malignant characteristics. Hu et al. (17) demonstrated that EGCG markedly suppressed tumor-induced HIF-1 expression, thereby inhibiting tumor proliferation.

These observations are consistent with those of Suhail and colleagues who further substantiated the impact of EGCG on Nuclear factor-kappa B (*NF-κB*) activity in PC cell lines. *NF-κB*, recognized as a therapeutic target across diverse cancers, plays a pivotal role in inflammatory responses. With increasing concentrations of EGCG (10–100 μM) for 24 h. A significant reduction in the viability and proliferation of MIAPaCa-2 and SU.86.86 cells was observed with increasing EGCG concentrations, confirming the influence of EGCG on *NF-κB* activity in PC cells (18).

These findings align with those reported by Suhail et al. (19), who further corroborated the inhibitory effects of EGCG on *NF-κB* activity in PC cell lines. *NF-κB* identified as a critical therapeutic target in various malignancies, plays a central role in mediating inflammatory responses. A pronounced decrease in the viability and proliferation of MIAPaCa-2 and SU.86.86 cells was observed with escalating EGCG concentrations (10–100 μM) over 24 h, further affirming EGCG’s modulatory impact on *NF-κB* activity in PC cells.

*In vitro* data revealed the efficacy of EGCG in diminishing cell growth, fostering apoptosis, and suppressing *NF-κB* activity in the probed cell lines. Additionally, EGCG suppressed the expression of *NF-κB* target genes. In combination with biomaterials, Cunha and associates (20) reported that EGCG might increase gemcitabine sensitivity, moderately obstructing the Akt pathway to curtail the migration, invasion, and epithelial–mesenchymal transition of PC cells. Liu et al. (21) proposed the importance of O-like forkhead transcription factors (*FOXOs*) in cell proliferation, angiogenesis, metastasis, and tumorigenesis. Empirically, EGCG inhibits *in situ* tumor growth, angiogenesis, and metastasis in PC, which is associated with *PI3K/AKT* and ERK pathway suppression coupled with *FKHRL1/FOXO3a* activation (22).

#### 2.2.2 Apigenin

Apigenin (4′,5,7-trihydroxyflavone) (Figure 1B), which is categorized as a *flavone*, is a naturally occurring compound derived from plants. It serves as the aglycone for multiple glycosides. Apigenin has a molecular formula of C_15_H_10_O_5_ and a molecular weight of approximately 270 g/mol. Its attributes include antioxidant, anti-inflammatory, anti-cancer, antigenotoxic, antiallergic, neuroprotective, cardioprotective, and antibacterial effects (23, 24). Recently, Apigenin has gained prominence as a health-promoting agent that is distinct from other structurally related flavonoids because of its minimal toxicity and notable impact on normal cells in contrast to cancerous cells (25, 26).

Feng et al. (27) revealed that at a suitable concentration (2.5–10 μM for 72 h), Apigenin has the capacity to increase Bcl-2 expression while decreasing Bax expression at both the gene and protein levels. Additionally, it promotes the proliferation of NK cells and amplifies their cytotoxicity through the activation of the JNK and ERK pathways (27).

Furthermore, regarding the restraint of tumor cell proliferation, Wu et al. (28) investigated the influence of *NF-κB* on the curbing of Apigenin-induced PC cell growth. Wu et al. (28) revealed that Apigenin effectively inhibits IκB kinase (*IKK*) activity, thereby increasing the anti-cancer potency of Apigenin. Notably, the overexpression of *IKK* mitigated the Apigenin-induced inhibition of cell growth. Moreover, the administration of Apigenin (30 mg/kg) inhibited PC progression and *IKK* activation in xenografts of nude mice. These findings collectively imply that Apigenin is promising for impeding *IKK*-mediated activation of *NF-κB* (28).

Moreover, Apigenin influences molecular pathways such as *HIF, GLUT-1*, and *VEGF*, thereby disrupting the proliferation and malignancy of PC cells (29). To promote cancer cell proliferation, Apigenin not only triggers apoptotic cell death through increased reactive oxygen species (ROS) generation but also orchestrates the downregulation of anti-apoptotic factors such as *Bcl-2* and *Bcl-xl*, accompanied by the upregulation of apoptotic elements such as Bax and Bim (30), which have antiproliferative effects on cancer cells. Additionally, Apigenin orchestrates cell cycle arrest at the G2/M and S phases. To curb cancer cell metastasis, Apigenin affects the PI3K/Akt/mTOR signaling cascade and regulates the expression of *MMP-9* (31), a pivotal contributor to the progression and invasiveness of cancer cells (32).

#### 2.2.3 Baicalein

Baicalein, a flavonoid extract (5,6,7-trihydroxyflavone) (Figure 1C) originating from the desiccated root of *Scutellaria baicalensis Georgi* (33), is a prominent bioactive component within the traditional herbal remedy recognized as the *Chinese skullcap* (or Huang Qin). It is extracted from the roots of this plant along with other flavonoids, such as Baicalin, Wogonin, and Nor Wogonin (34). Baicalein has a spectrum of pharmacological effects, encompassing anti-inflammatory, antioxidant, and antiviral actions, as well as cardiovascular protection, and has remarkable anti-cancer potential (35, 36).

The main molecular mechanisms underlying Baicalein’s anti-tumor effects include CDK-dependent cell cycle inhibition, scavenging of ROS, moderation of the MAPK/Akt/TOR pathways, and induction of apoptosis through the activation of caspase-9/−3. Additionally, it suppresses cancer metastasis and invasion by downregulating the expression of the metalloproteinases MMP-2/−3 (37).

Ma et al. scrutinized the mechanisms underlying Baicalein’s efficacy in both *in vivo* and *in vitro* treatment of PC (38). These findings reveal the potential of Baicalein to curtail the proliferation of Panc-1 PC cells, thereby promoting their apoptosis. It also highlighted the influence of Baicalein on the miRNA landscape within PC cells and revealed that 20 and 39 miRNAs were accordingly up- and downregulated, respectively, in Panc-1 cells exposed to 100 μM Baicalein compared with those in the control group. Subsequent validation revealed that miR-139-3p and miR-196b-5p drive apoptosis in human Panc-1 PC cells by targeting the yeast gene *NOB1* and the growth inhibitory factor *ING5*, respectively (39).

Parallel investigations suggest that Baicalein has regulatory effects on the inflammatory microenvironment of tumors. This is twofold: first, by suppressing *NF-κB* activation in pancreatic acinar cells triggered by tumor necrosis factor-*α*; second, by inhibiting *TNF* secretion from activated macrophages (40). This orchestrated modulation of the inflammatory milieu contributes to the anti-PC role of Baicalein (41). Zhou et al. (42) revealed that further underscores the versatility of Baicalein. It effectively impedes PC proliferation and invasion by repressing the expression of the oncogene *NEDD9* and inactivating the *Akt* and *ERK* cascades (42). Additionally, Baicalein has the ability to prevent the proliferation of human PC stem cells. This is achieved through the inhibition of the secretory glycoprotein factor SHH signaling pathway, culminating in pancreatic CSC apoptosis and the inhibition of CSC self-renewal (43).

Baicalein demonstrates specific anti-cancer effects against pancreatic ductal adenocarcinoma (PDAC), a distinct subtype of PC characterized by unique pathological features. While various forms of PC, including PDAC, display different biological behaviors and treatment responses, Baicalein’s mechanisms are notably selective toward PDAC. It inhibits the enzymatic activities of 5-lipoxygenase (5-LOX) and 12-lipoxygenase (12-LOX), thereby reducing cellular proliferation. Additionally, Baicalein promotes apoptosis in PDAC cells by inducing Cyt-c release and increasing the *Bax/Bcl-2* protein ratio, further facilitating programmed cell death (44). In addition, Baicalein plays a key role in inhibiting the expression of the *Mcl-1* protein and its corresponding mRNA, overcoming chemotherapy resistance in PC cells. It significantly inhibits PC cells proliferation in a dose-dependent manner, with the 5% concentration of Baicalein extract showing notable effects (45).

#### 2.2.4 Wogonin

Wogonin (Figure 1D), a flavonoid derived from the root of *Scutellaria baicalensis*, has demonstrated significant anti-cancer efficacy across various malignancies, including PC. Liu et al. (46) demonstrated that Wogonin exerted a potent inhibitory effect on the survival and proliferation of PC cells, ultimately leading to cell death. Mechanistically, Wogonin induced ferroptosis, a distinct and highly regulated form of cell death characterized by iron-dependent lipid peroxidation. Ferroptosis is initiated by the depletion of intracellular glutathione and the subsequent inactivation of glutathione peroxidase 4 (GPX4), leading to oxidative damage and cell death (47). Wogonin increased Fe^2+^ levels, lipid peroxidation, and superoxide production while concurrently downregulating the expression of ferroptosis suppressor genes and reducing glutathione levels in PC cells (48). Importantly, the Wogonin-induced ferroptosis-associated processes were ameliorated by ferroptosis inhibitors, confirming the central role of ferroptosis in its anti-cancer activity. Furthermore, treatment with Wogonin, in combination with ferroptosis inducers, significantly amplified ferroptosis-related processes (49). These findings highlight the potential of Wogonin as a therapeutic agent in PC, primarily through its ability to trigger ferroptosis via the Nrf2/GPX4 signaling axis (46).

#### 2.2.5 Chrysin

Chrysin (Figure 1E) is a naturally occurring compound endowed with anti-inflammatory, antioxidant, and anti-cancer properties. In the context of this investigation, Chrysin activated the G protein-coupled estrogen receptor (GPER), resulting in arrest of the PC tumor cell cycle and a reduction in viability. Notably, Chrysin-treated tumors presented diminished levels of ROCK1, TAGLN2, and FCHO2, indicating that Chrysin inhibits PC progression (50). Recent studies have shown that the induction of ROS contributes to the anti-tumor mechanism of gemcitabine. In this spectrum, human carbonyl reductase 1 (CBR1) has emerged as a safeguard against oxidative harm. High expression of CBR1 in PC tissues is correlated with clinicopathological features. Chrysin intervenes by inhibiting CBR1, thereby increasing ROS levels and inducing ferroptosis in PC cells. Ferroptosis, a regulated form of cell death that is critically dependent on iron and lipid peroxidation, is distinct from apoptosis or necrosis. By promoting ferroptosis, Chrysin not only increases cancer cell death but also enhances the sensitivity of PC cells to gemcitabine, offering a promising therapeutic strategy through the dual targeting of CBR1 and ferroptosis in PC (51).

#### 2.2.6 Quercetin

Quercetin (Figure 1F), a polyphenolic flavonoid belonging to the category of *flavonols*, is widely present in everyday foods, including plants, vegetables, nuts, seeds, fruits, tea, and red wine (52, 53). Quercetin has a distinctive flavonoid structure (C6-C3-C6 backbone) characterized by two benzene rings linked by a 3-carbon heterocyclic pyrone. Quercetin has the capacity to impede or stimulate various biological processes, including autophagy, apoptosis, and cell growth; reduce or inhibit epithelial–mesenchymal transition (EMT) and oxidative stress; and augment susceptibility to chemotherapy agents and is used as an adjuvant for PC treatment or as an alternative drug to relieve PC (54).

Guo et al. (55) investigated the therapeutic potential of Quercetin in the sonic hedgehog (SHH) signaling pathway in pancreatic ductal adenocarcinoma (PDAC). These findings suggest that Quercetin curtails PC cell proliferation through the suppression of c-Myc. Additionally, it inhibits epithelial–mesenchymal transition (EMT) by diminishing TGF-β1 levels, which, in turn, inhibits migration and invasion. Notably, Quercetin (100 μM) dampened sonic hedgehog (SHH) activity, resulting in diminished PDAC growth and metastasis in a nude mouse model (55).

Lan et al. (56) reported that cell death was exacerbated in a dose-dependent manner by Quercetin treatment of MIA Paca-2 GEMR cells at 24 h or 48 h, demonstrating that Quercetin can accelerate the death of human PC cells, inhibit the PI3K/Akt/mTOR pathway, and increase the sensitivity of human PC cells to chemical drugs. Yu et al. (57) reported that Quercetin (0, 20, 40, or 80 μM for 24 h) can reduce the release of matrix metalloproteinases (MMPs) in human PC cells and activate STAT3/IL. Studies have shown that it can impact the expression of the MMP; human PC cells stimulate the STAT3 signaling pathway through the secretion of the MMP and epithelial–mesenchymal transition, whereas Quercetin reverses IL-6-induced EMT and PC cells invasion. Furthermore, Nwaeburu et al. (58) investigated the effect of Quercetin on miRNA expression in PC cells. Studies have shown that the combination of gemcitabine and Quercetin, by loading gemcitabine and Quercetin in biodegradable lactic acid–coglycolic acid (CO-glycolic acid) nanoparticles (NPs), can play a targeted role and synergistically inhibit the migration of PC cells (59).

#### 2.2.7 Rutin

Rutin, chemically known as 3,30,40,5,7-pentahydroxyflavone-3-rhamnoglucoside (Figure 1G), is a flavonoid predominantly found in medicinal plants like apples and tea. This natural polyphenolic compound exhibits diverse pharmacological activities, including antihypertensive, cardioprotective, neuroprotective, and anticancer effects, attributed to its capacity to modulate biological pathways and its antioxidant properties. Research demonstrates that rutin lowers blood pressure by enhancing endothelial function and reducing oxidative stress (60). Its cardioprotective effects are associated with decreased inflammation and oxidative damage (61). In neurodegenerative models, rutin mitigates neuronal damage and enhances cognitive function (62). Furthermore, it induces apoptosis in cancer cells and inhibits tumor growth, underscoring its potential as a therapeutic agent for various health conditions (63).

Huo, M., et al. employed three distinct PC cell lines as experimental models to investigate the anti-PC potential of Rutin. Cell viability assessments, wound healing migration assays, and apoptosis assays revealed the potent antiproliferative, antimigratory, and proapoptotic properties of Rutin. These findings demonstrated that Rutin facilitated the induction of apoptosis in PC cells. Notably, Rutin (10 μg/mL) substantially increased the expression of miR-877-3p, concurrently suppressing Bcl-2 transcription and consequently promoting the apoptosis of PC cells (64).

#### 2.2.8 Fisetin

Fisetin (3,7,30,40-tetrahydroxyflavone) (Figure 1H) is a naturally occurring dietary polyphenolic flavonol that is known to induce a wide range of potential bioactivities, such as antioxidant, anti-cancer, and anti-inflammatory effects, in diverse cancer types. The mechanisms through which Fisetin orchestrates epigenetic regulation in cancer have not been fully elucidated. In PDAC, effective chemotherapy is impeded by DNA damage repair. Fisetin has emerged as a potential promising anti-tumor agent that triggers DNA damage. Huang et al. (65) demonstrated that Fisetin induces DNA double-strand breaks (DSBs) while simultaneously suppressing homologous recombination (HR) repair mechanisms via m6A modification in PDAC cells. Moreover, Xiao, Y., et al. have revealed the capacity of Fisetin to prevent PC by specifically targeting the PI3K/AKT/mTOR signaling cascade while sparing the JAK2 cascade (66). Fisetin emerged as an agent capable of suppressing cell proliferation, inducing DNA damage, and promoting S-phase arrest in PDAC. The effects of Fisetin include the upregulation of RFXAP and other DNA damage response genes. Further insights revealed the potential of Fisetin to increase the efficacy of chemotherapy in PC cells. These findings suggest that Fisetin inhibits PDAC proliferation by inducing DNA damage through RFXAP/KDM4A-dependent histone H3K36 demethylation (67).

Jia et al. (68) demonstrated that substantiates the *in vivo* anti-tumor impact of high concentrations of Fisetin (200 μM) on PC by leveraging a mouse xenograft model. Intriguingly, Fisetin treatment enhanced the AMPK/mTOR signaling pathway. However, the introduction of the AMPK inhibitor compound C failed to attenuate autophagy. The inhibition of the AMPK/mTOR pathway via the AMPK inhibitor compound C seems to increase p8-dependent autophagy. This finding reveals a potential interplay between the AMPK/mTOR and p8-dependent pathways (68).

The death receptors of the tumor necrosis factor (TNF) receptor superfamily exhibit constitutive NF-kB activation in PC cells. Murtaza et al. (69) revealed the dual effects of Fisetin on inducing apoptosis and restraining the invasion of chemotherapy-resistant AsPC-1 PC cells. It achieves this by impeding DR3-mediated NF-kB activation.

#### 2.2.9 Myricetin

Myricetin (3,5,7,30,40,50-hexahydroxyflavone) (Figure 1I) is an important flavonoid that is chiefly found in glycoside forms (O-glycosides) in fruits, vegetables, berries, nuts, herbs, plants, beverages and medicinal plants. By utilizing primary and metastatic cell lines derived from PC, Phillips et al. (70) have shown the capacity of the flavonoid Myricetin to prompt cell death via apoptosis in PC cells. This phenomenon is accompanied by a notable reduction in PI3 kinase activity within an *in vitro* setting. Notably, when applied *in vivo*, Myricetin (12.5–200 μM for 24 h) treatment of orthotopic pancreatic tumors led to tumor regression and curtailed metastatic dissemination. Notably, the lack of toxicity of Myricetin is evident both *in vitro* and *in vivo*, highlighting its potential as an efficacious therapeutic approach for treating PC (70).

#### 2.2.10 Kaempferol

Kaempferol (3,5,7-trihydroxy-2-(4-hydroxyphenyl)-4H-chromen-4-one) (Figure 1J), also known as indigo yellow, is a flavonoid compound that is present extensively in various vegetables, fruits, and herbal medicines. Kaempferol has various bioactive functionalities, including antioxidant, anti-inflammatory, anti-apoptotic, and anti-cancer properties. Zhang et al. (71) has documented the efficacy of Kaempferol in conjunction with erlotinib (ERL), an epidermal growth factor receptor (EGFR) tyrosine kinase inhibitor (TKI), for preventing the growth of PC. Nonetheless, the clinical effectiveness of ERLs has encountered limitations owing to the activation of alternative pathways that circumvent EGFR signaling. Studies have shown that KAE may be an effective therapeutic candidate for sensitizing PC cells to ERL through the inhibition of PI3K/AKT and EGFR signaling (71). From the perspective of antioxidant mechanisms, Kaempferol exerts its anti-cancer effects by effectively scavenging ROS. As demonstrated by Wang et al. (72) Kaempferol (25–50 μM) stimulates apoptosis *in vitro* by increasing ROS production, a phenomenon intricately interlinked with the Akt/mTOR signaling pathway.

#### 2.2.11 Naringenin

Naringenin (2,3-dihydro-5,7-dihydroxy-2-(4-hydroxyphenyl)-4H-1-benzopyran-4-one) is a flavanone (Figure 1K) (73). Major dietary sources of Naringenin include grapefruit, lemon, orange, and tomato (74). In studies where Naringenin was administered to AsPC-1 and PANC-1 PC cell lines, it was shown to significantly downregulate epithelial–mesenchymal transition (EMT) markers, primarily through modulation of the TGF-β1/Smad signaling pathway. This intervention not only inhibited cancer cell invasiveness but also effectively attenuated gemcitabine resistance (75–77).

Hesperidin, another flavonoid frequently found in citrus fruits, is often combined with Naringenin due to its synergistic effects in cancer treatment. When administered together, Naringenin and Hesperidin activated caspase-3, resulting in a significant inhibition of human PC cell proliferation, a result that was not observed with individual treatments alone. This combination also suppressed cancer cell migration while sparing normal cells from damage. In comparison to the individual treatments, the εCUP mimic, a synthetic analog designed to replicate key functional properties of the target protein, significantly inhibited the phosphorylation of FAK and p38, both of which are critical for cancer cell survival and proliferation. Moreover, the εCUP mimic exhibited a strong *in vivo* anti-proliferative effect on xenograft models (*p < 0.001*), demonstrating its potential as a promising and non-toxic therapeutic strategy for PC in combination with Naringenin (7.5 μM) and Hesperidin (2.5 μM) (75).

#### 2.2.12 Isoliquiritigenin

Isoliquiritigenin (Figure 1L) is a derivative of liquorice chalcone; its molecular composition is C_15_H_12_O_4_, and its molecular weight is 256.26. It is scientifically known as (E)-1-(2,4-dihydroxyphenyl)-3-(4-hydroxyphenyl)propan-2-en-1-one (78) and is derived from the root of liquorice. Elevated autophagy and ROS accumulation are hallmarks of the advancement and progression of PC (79–81). In view of this background, targeting autophagy by pharmacological means has become a promising therapeutic approach for treating PC (82). Notably, Zhang et al. (83) have extensively demonstrated the inhibitory impact of Isoliquiritigenin on the *in vitro* and *in vivo* growth of PC cells. These findings revealed that Isoliquiritigenin treatment increased the expression and accumulation of LC3-II and p62 in different manners. Autophagy, a cellular mechanism for degrading and recycling components, can be monitored using the RFP-GFP-LC3 system, which differentiates early autophagosomes from late autolysosomes. This fluorescent marker system revealed that Isoliquiritigenin functions as a late-stage autophagy inhibitor, significantly enhancing the therapeutic effects of gemcitabine and 5-fluorouracil in pancreatic cancer cells through synergistic inhibition of cell survival mechanisms. Furthermore, Isoliquiritigenin treatment increased the presence of CD4+ and CD8+ T cells within tumor tissues, suggesting that Isoliquiritigenin have the potential to trigger protective anti-tumor immune responses. Zhang et al. (83) also highlights how Isoliquiritigenin-induced activation of p38 MAPK impedes autophagy, thus antagonizing autophagy induced by p38 inhibitors. These findings underscore the potential of Isoliquiritigenin as anti-cancer agents, enhancing the efficacy of traditional chemotherapeutic agents such as GEM and 5-FU in the treatment of PC.

#### 2.2.13 Xanthohumol

Xanthohumol (84) (Figure 1M), classified as a prenylated chalcone, serves as the primary flavonoid present in the hop plant (*Humulus lupulus* L.). Extensive research has demonstrated the ability of Xanthohumol to hinder the proliferation of diverse human cancer cell types, including PC (85).

Cykowiak et al. (86) demonstrated that Xanthohumol, phenylethyl isothiocyanate, resveratrol, indole-3-carbinol, and their respective combinations can upregulate the expression of *Nrf2* and antioxidant enzymes. Additionally, they investigated the combined effects of Xanthohumol and phenylethyl isothiocyanate (PEITC) on PC cells. In murine models, the combination of Xanthohumol and PEITC was found to inhibit tumor growth, potentially through the modulation of the NF-κB, JAK/STAT, or PI3K signaling pathways (86).

Xanthohumol has multiple functions, triggering caspase-dependent and caspase-independent apoptosis while also inhibiting invasion and angiogenesis (87). PC is dependent on angiogenesis for invasive growth and metastasis (88). Xanthohumol acts as an inhibitor; *in vitro*, it blocks PC cell proliferation at concentrations >10 μmol/L and specifically targets NF-kB-driven angiogenesis at concentrations <5 μmol/L without causing cytotoxicity. Downregulation of NF-kB p65 and angiogenic factors (VEGF, IL-8) *in vivo* confirmed its role in attenuating PC-induced angiogenesis by blocking NF-kB.

Saito et al. (87) demonstrated that the inhibition of angiogenesis by Xanthohumol mainly targeted NF-kB and downstream elements, even at low concentrations (>10 μmol/L). *In vitro* and *in vivo* data highlight the ability of Xanthohumol to improve PC cell proliferation and tumorigenesis through cell cycle inhibition, G2/M phase arrest, and induction of apoptosis, which echoes similar findings in other cancer cell studies.

Jiang et al. (89) revealed that Xanthohumol induced cell cycle arrest and apoptosis in PC cells by inhibiting STAT3 phosphorylation, identifying STAT3 signaling as a key therapeutic target. Kunnimalaiyaan et al. (85) revealed that the effect of Xanthohumol on PC cell lines was evaluated, resulting in increased apoptosis due to Notch1 pathway inhibition, as indicated by decreased Notch1, HES-1 and survivin levels. This effect was further supported by the downregulation of Notch1 promoter activity. The role of the reduction in Notch1 is highlighted. These results suggest that the multifaceted effects of Xanthohumol are expected to inhibit angiogenesis and PC growth through the NF-kB and Notch1 pathways, providing a potential avenue for therapeutic intervention.

#### 2.2.14 Silibinin

Silibinin (Figure 1N), a natural flavonoid derived from the milk thistle plant *Silybum marianum*, has emerged as a promising candidate in the fight against PC (90). Lee et al. (91) investigated the potential anti-cancer pathway of Silibinin by modulating JNK/MAPK signaling.

Silibinin permeabilizes the mitochondrial outer membrane and subsequently releases apoptosis-inducing factors via the downregulation of anti-apoptotic proteins (*Bcl-2* and *Bcl-xl*) and the upregulation of proapoptotic proteins (*Bax* and *Bim*). Bai et al. (92) revealed that decreased expression of *Bcl-2* and *Bcl-xl*, elevated Bax and *Cyt-c* expression, reduced ATP production, increased ROS generation, and cellular damage collectively contribute to mitochondrial apoptosis. In their *in vivo* xenograft model, a reduction in the expression of *Bcl-2* and *Bcl-xl*, along with an increase in the expression of *Bax* and *Cyt-c*, decreased ATP production and increased ROS production and cellular damage, together leading to mitochondrial apoptosis (92).

In an *in vivo* xenograft model, inhibition of the *ERK* protein by Silibinin, administered at IC₅₀ (the concentration required to achieve 50% inhibition) and 2 × IC₅₀ (twice the concentration required to achieve 50% inhibition) for 48 h, triggered a reduction in the mitochondrial membrane potential and subsequent *Cyt-c* release, ultimately driving apoptosis in PC cells.

Additionally, the effects of Silibinin on human PC cell lines (BxPC-3 and PANC-1) were comprehensively evaluated across various stages of disease progression, both *in vitro* and *in vivo*. PC cell line SW1990 have shown that Silibinin enhances cell viability, induces apoptosis, modulates ROS levels, affects ATP production, and stimulates autophagy, suggesting its multifaceted effects on mitochondrial function and cellular processes (93).

Feng et al. (94) extended their research to combination therapy, where the synergistic combination of the histone deacetylase (HDAC) inhibitor TSA and Silibinin induced G2/M cell cycle arrest and apoptosis in the cell lines Panc1 and Capan2. The treatment also downregulated the expression of key cell cycle regulators (*cyclin A2, cyclin B1/Cdk1*) and survivin. These findings suggest that the innovative combination of HDAC inhibitors and Silibinin has the potential to treat PC (94).

#### 2.2.15 Puerarin

Puerarin (Figure 1O) (8-(*β*-D-glucopyranosyl)-4′,7-dihydroxyisoflavone) is a natural isoflavone extracted from the traditional Chinese medicine Puerarin that has the capacity to trigger apoptosis and impede proliferation, thus harboring promising anti-tumor attributes. The tumor-suppressive effects of Puerarin were evaluated through a series of comprehensive *in vitro* and *in vivo* experiments. In a xenograft mouse model of PDAC, treatment with various concentrations of Puerarin for 24 h markedly restrained the proliferation of PC cells. This effect is achieved by eliciting mitochondria-dependent apoptosis within the PC cells, which is mediated through disruption of the *Bcl-2/Bax* equilibrium. Furthermore, Puerarin inhibited PC cells migration and invasion by antagonizing epithelial–mesenchymal transition (EMT). Puerarin administration reduces PDAC growth and metastasis in a nude mouse model. Mechanistically, Puerarin exerts therapeutic effects on PDAC by inhibiting Akt/mTOR signaling. Further studies revealed that mTOR activators abolished the inhibitory effect of Puerarin on PC cells, suggesting that mTOR plays a crucial role in the anti-tumor effect of Puerarin on PDAC. Puerarin hinders glucose uptake and metabolism by diminishing the oxygen consumption rate (OCR) and extracellular acidification rate (ECAR), effects governed by HIF-1α and the glucose transporter *GLUT1*. These findings suggest that the suppression of glucose uptake and metabolism by Puerarin can be attributed to its regulation of Akt/mTOR activity, indicating its potential therapeutic application in treating pancreatic ductal adenocarcinoma (PDAC) (95).

#### 2.2.16 Icariin

Icariin (Figure 1P) is derived from *Epimedii*, a traditional Chinese medicinal herb (96) represents a principal biologically active constituent. In the chemical arrangement of 8-prenyl flavonoid glycosides, Icariin is a light yellow powder with the molecular formula C_33_H_40_O_15_ (97). Pharmacological studies has confirmed the neuroprotective, cardioprotective, anti-inflammatory, and anti-cancer effects of Icariin (98, 99).

Wang et al. (99) have demonstrated that Icariin, with a purity of 30.54%, can be isolated from *Herba Epimedii*, and its direct cytotoxic effects on PC cells have been confirmed through *in vivo* experiments. At concentrations of 100 μM and 150 μM, Icariin showed significant efficacy against epithelial pancreatic tumors (100). Moreover, Icariin reduces the infiltration of PMN-MDSCs (polymorphonuclear myeloid-derived suppressor cells) into tumors and decreases their count in the spleen. PMN-MDSCs are a subset of myeloid-derived suppressor cells characterized by a granulocytic phenotype. These cells play a crucial role in suppressing anti-tumor immunity by inhibiting T cell activity and promoting tumor growth through immune evasion mechanisms. This is attributed to the reduction in the proportions of M2 macrophages and PMN-MDSCs in tumor and spleen tissues caused by Icariin, potentially suppressing tumor growth. Mechanistically, Icariin impedes the polarization of RAW264.7 cells, a murine macrophage cell line commonly used in immunological research, into M2 macrophages by downregulating the STAT6 pathway. Zheng et al. (100) revealed that Icariin hinders PC progression through Panc02 cell apoptosis, migration inhibition, and immune microenvironment modulation involving M2 macrophages and PMN-MDSCs (via STAT6 downregulation). In summary, Icariin has emerged as a prospective anti-tumor and immunomodulatory agent for treating PC.

Omura et al. (101) indicates that miR-9-5p downregulation in PC tissues and cell lines is linked to shorter patient survival. Thus, increasing the level of miR-9 has the potential to restrain PC development. Amidfar et al. (102) probed the effects of Icariin on PC proliferation and apoptosis via *in vitro* cell culture. They observed reduced clone counts in PC BxPC-3 cells, alongside decreased expression of the proliferation markers PCNA and Ki67.

In a normal system, cell proliferation and apoptosis maintain equilibrium. Amidfar et al. (102) demonstrated that Icariin promoted apoptosis in BxPC-3 PC cells, increasing the expression of Bax and cleaved caspase-3 while downregulating the expression of the apoptosis marker Bcl-2. Caspase-3 plays a pivotal role in the execution of apoptosis. Bcl-2 inhibits Cyt-c release and caspase-3 activation, counteracting the proapoptotic Bax gene and inhibiting apoptosis (102). Consequently, Icariin promotes apoptosis in BxPC-3 PC cells. Furthermore, Huang et al. (103) revealed that Icariin augments miR-9 expression in BxPC-3 PC cells. Suppressing miR-9 reversed the effects of Icariin on cell proliferation and apoptosis. Notably, the positive control drug cisplatin had no effect on miR-9 expression in these cells. These findings suggest distinct mechanisms between cisplatin and Icariin, despite their similar effects on PC BxPC-3 cell proliferation and apoptosis. Icariin elevates miR-9 to inhibit cell proliferation and induce apoptosis in these cells (103).

In conclusion, Icariin inhibits cell proliferation and promotes apoptosis in BxPC-3 PC cells, which is associated with miR-9 upregulation. Dai et al. utilized a nude mouse xenograft model in which various doses of Icariin and amphotericin B were administered via intraperitoneal injection. Different Icariin doses led to reduced *β*-catenin and phosphorylated *NF-κB* p65 protein expression, suggesting the potential of Icariin to control subcutaneously transplanted PC tumor growth and mobility via Wnt/β-catenin and *NF-κB* pathway inhibition.

#### 2.2.17 Morusin

Morusin (Figure 1Q), a flavonoid isolated from *Morus alba* (mulberry tree), has demonstrated potent anti-cancer properties by inhibiting key oncogenic pathways such as nuclear factor kappa B (NF-κB) and signal transducer and activator of transcription 3 (STAT3) in prostate, pancreatic, and liver cancers (104). Aberrant *NF-κB* activation is closely linked to tumor progression, inflammation, and chemoresistance in PC, making it a critical target for therapeutic intervention.

**Figure 2:**
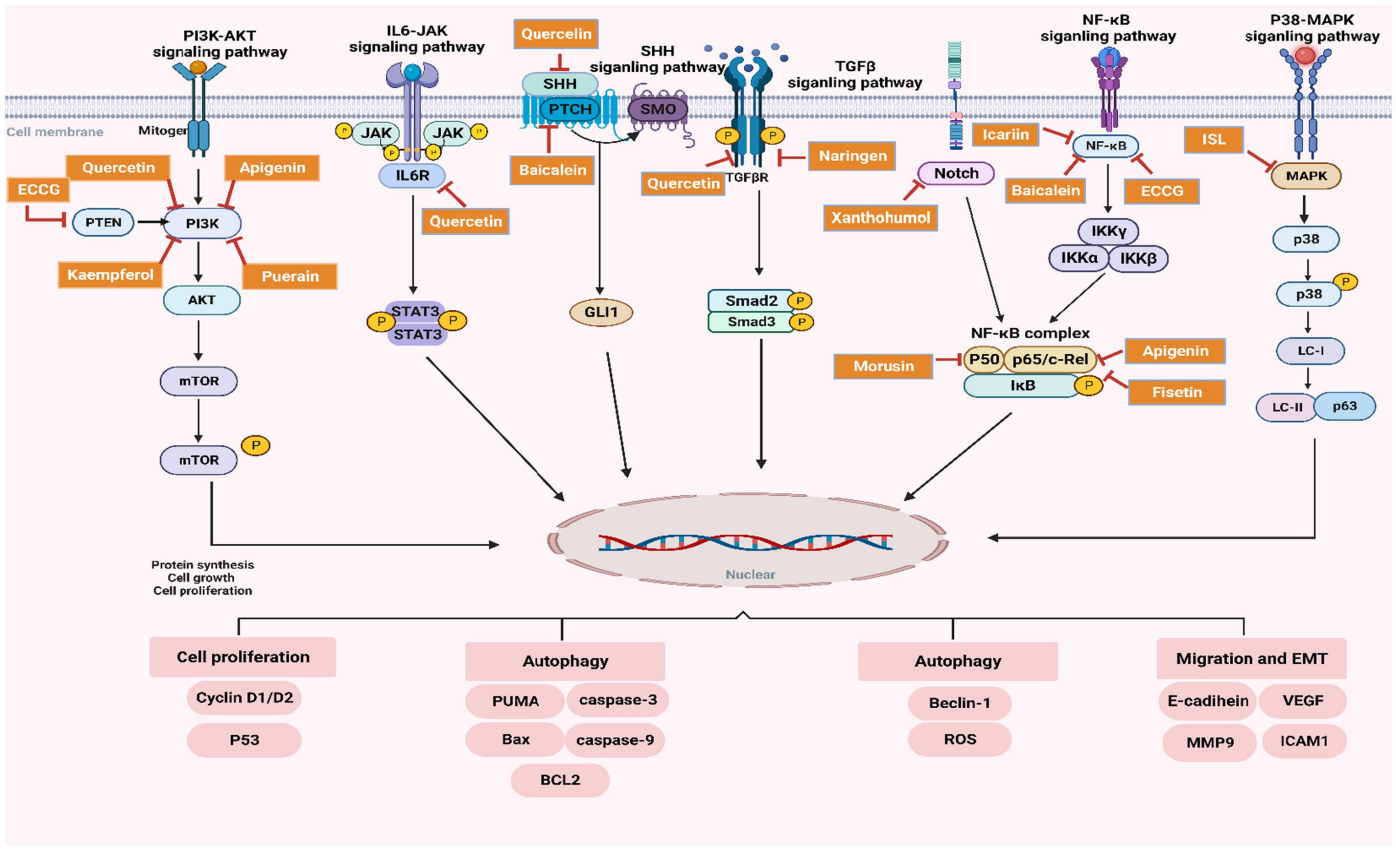
Schematic representation of the regulatory effects of flavonoids on key signaling pathways in PC.

Morusin specifically inhibits IκB kinase (*IKK*), preventing the phosphorylation and degradation of IκB*α*, the endogenous inhibitor of *NF-κB* (as shown in Figure 2). This stabilization of IκBα sequesters the *NF-κB* complex (*p50/p65*) in the cytoplasm, thereby blocking its nuclear translocation and transcriptional activity. As a result, Morusin suppresses the expression of *NF-κB* target genes, including pro-inflammatory cytokines (e.g., *TNF-α, IL-6*), anti-apoptotic proteins (e.g., *Bcl-2, Bcl-xL*), and oncogenes (e.g., *Cyclin D1, MMP-9*, and *VEGF*) that drive tumor growth, proliferation, and metastasis.

Preclinical models have demonstrated that Morusin significantly reduces PC cell viability, inhibits tumor invasiveness, and promotes apoptosis by downregulating these *NF-κB*-mediated oncogenic pathways. Figure 2 illustrates Morusin’s mechanism of action, highlighting its role in inhibiting the phosphorylation of *IκBα*, preventing *NF-κB* nuclear translocation, and thereby disrupting the transcriptional regulation of genes critical for PC progression.

#### 2.2.18 Anthocyanins

Anthocyanins (Figure 1R), particularly anthocyanin-3-glucoside (C-3-O-G), are well-known flavonoid compounds with potent anti-cancer chemopreventive properties. Research by Mostafa, H., et al. has thoroughly investigated the effects of plasma-isolated anthocyanins and their metabolites on PC cell migration, revealing critical molecular targets involved in cancer progression (105).

In their study, anthocyanin-rich juice was administered to 35 healthy participants over a 28-day trial, compared to a placebo group. Plasma metabolites were extracted both before and after the trial using solid-phase extraction. The subsequent migration studies demonstrated a marked reduction in PANC-1 cell migration, thereby highlighting the efficacy of anthocyanins in inhibiting cancer cell movement. Furthermore, the expression of ß1- and ß4-integrins, as well as intercellular adhesion molecules, was significantly reduced following the intake of the anthocyanin-rich juice, suggesting a direct impact on cellular adhesion mechanisms.

Moreover, pooled plasma from the most responsive group demonstrated the ability to inhibit key cancer signaling pathways, such as *NF-kB-p65* and focal adhesion kinase, in both cancerous and endothelial cells. This is important because *NF-kB-p65* orchestrates cancer progression and inflammation, while focal adhesion kinase maintains metastasis in the later. These findings indicate that targeting these-signaling pathways has therapeutic potential for Polyphenol-rich Anthocyanin Mixtures (PAMs), which are natural compounds found in plants, known for their anti-cancer, anti-inflammatory, and antioxidant properties. PAMs show promise in treating metastatic malignancies (MM), which refers to advanced stages of cancer where the disease has spread from its original site, specifically in relation to PC. Further analysis on metabolomic study demonstrated that 14 distinct potential pathway-associated altered-metabolites were detected in four groups of o-coumaric acid, peonidin-3-galactoside abundance presented negative correlation here with migration of PANC-1 cells.

Consequently, these metabolites may serve as important biomarkers in monitoring the therapeutic efficacy of anthocyanin treatments.

In conclusion, the findings presented by Mostafa, H., et al. provide compelling evidence that PAMs could serve as a promising strategy for inhibiting PC cell migration and reducing metastasis. However, further research is necessary to elucidate the long-term effects of anthocyanins on cancer progression and to optimize their bioavailability for clinical applications.

## 3 Mechanism of flavonoids in the treatment of PC

### 3.1 Regulation of apoptosis, inflammation, and oxidative stress in PC by flavonoids

Flavonoids exert profound regulatory effects on key biological mechanisms in PC, including apoptosis, inflammation, and oxidative stress, as illustrated in detail in Table 1. Each of these processes is influenced by specific molecular targets and signaling pathways that flavonoid compounds interact with. The following subsections elaborate on these interactions and the role of flavonoids in modulating these mechanisms, with a summary of the key flavonoids and their targets provided in Table 1.

**Table 1:** Summary of flavonoids, their sources, mechanistic actions, and molecular targets in PC.

No.	Compound	CAS no.	Molecular weight	Source	Mechanism	Regulates	Regulated by	Binds	Ref
(a)	Epigallocatechin-3-gallate	989–51-5	458.38	Green tea	↓NF-κB ↓PI3K/AKT pathway	BAX, PTGS2, BCL2L1, NOS2, MMP2, MMP9, APP, TNF, Akt, ERK1/2, BCL2, CDKN1A, IL6, FOS, TP53	Epicatechin, Sod, CAT, tolcapone, entacapone, Quercetin, alpha-amylase	LEF, APP, Fibrinogen, VIM, NOTCH1, Adenosine Receptor, GABA-A receptor, TP53	(16–28)
(b)	Apigenin	520–36-5	270.23	Celery	↑NKcell、↓NF-κB、↑ROS、↓PI3K/AKTpathway	Vegf, MMP9, ICAM1, BCL2L1, BCL2, Ccl2, CASP3,NFkB(complex),TNFRSF10B,IL6, CXCL3,CCL3L3,PTGS2, VCAM1, CCL4	MUC1, benzyl 2-acetamido-2-deoxy-alpha-D-galactopyranoside, verlukast, SULT1A1, ABCG2	RPS9, ESR2, P glycoprotein, estrogen receptor, SLC25A5, ESR1, ABCG2, AHR, TNF	(29–38)
(c)	Baicalein	491–67-8	446.36	Baikal Skullcap Root	↓NF-κB、↓TNF-α ↓NEDD9、↑SHH pathway	BCL2, BAX, NOS2, Vegf, HMOX1, Akt, reactive oxygen species, IL6, KDR, MMP1, CASP3, ERK, lipid, RBL1, DDIT3	–	Estrogen receptor, SNCA	(39–51)
(d)	Wogonin	632–85-9	284.26	*Scutellaria baicalensis*	↓Nrf2/GPX4	BCL2, BAX, NOS2, Vegf, HMOX1, Akt, reactive oxygen species, IL6, KDR, MMP1, CASP3, ERK, lipid, RBL1, DDIT3	–	Estrogen receptor, SNCA	(52–55)
(e)	Chrysin	480–40-0	254.23	Seeds of *Bignoniaceae* wood butterfly	↑GPER、↓CBR1、ROS → Autophag、Ferroptosis	MUC5AC, CYP1A1, HMOX1, Vegf, reactive oxygen species, FLT1, transcription factor, ICAM1, SELE, PDE5A, NT5E, CXCL1, ADORA2B, IL6, STAT3	Tecto Chrysin, Apigenin, 3-methylcholanthrene, oltipraz, luteolin, acacetin, 6-hydroxyflavone, 7-hydroxy-5-methylflavone, diosmetin	P glycoprotein, CSNK2A1, CSNK2B, CSNK2A2, ESR2	(56, 57)
(f)	Quercetin	117–39-5	302.23	*Ginkgo biloba* extract	↓SHH↑TGF-β1/Smad2/3↓MMP、↓PI3K/Akt/mTOR pathway↑STAT3/IL-6 pathway	TNF, HMOX1, Hsp70, TP53, HIF1A, CASP3, AR, nitric oxide, Vegf, Akt, NFE2L2, IL6, MMP9, MUC5AC, BCL2L1	LCT, ethylenediaminetetraacetic acid, Kaempferol, verapamil, ABCG2, tocopherol, UGT1A4, D-glucose	P glycoprotein, ESR2, PI3K (complex), ESR1, Calcineurin, Calcineurin protein(s)	(58–65)
(g)	Rutin	153–18-4	610.51	Herb of Grace	↑ miR-877-3p↓ Bcl-2 transcription	NOS2, PTGS2, advanced glycation end-products, ROs, NA, D-glucose, Sod, CDH1, SOX2, CD44, Cellular Adhesion Molecule, NANOG, BCL2, MYC, Ca2+	N-nitro-L-arginine methyl ester, verapamil	P glycoprotein, Pdi, estrogen receptor	(66)
(h)	Fisetin	528–48-3	286.23	Coggygria	↓PI3K/AKT/mTOR pathway ↓NF-κB、↑RFXAP	BCL2L1, MMP9, NFKBIA, P38 MAPK, Vegf, TP53, BCL2, CCND1, RELA, Cbp/p300, Akt, FOS, cytokine, BAX, CDH1	–	SUMO1, estrogen receptor	(67–71)
(I)	Myricetin	529–44-2	318.23	*Myrica rubra*	↓PI3k pathway	Akt, PTGS2, MMP1, CYP2C9, P38 MAPK, nitric oxide, MAPT, rhodamine-123, NS5, CYP2D6, C, PI3K (complex), POLY, orf1ab, RNMT	Adenosine triphosphate, verapamil	PIM1, estrogen receptor, MAPT, FYN	(72)
(j)	Kaempferol	520–18-3	286.24	*Kaempferia galanga*	↓PI3K/AKT/mTOR pathway ↓NF-κB ↓ROS	Alp, BCL2L1, Akt, BCL2, TNF, PTGS2, CASP3, MMP9, CDKN1A, JAK3, PI3K (complex), CASP1, NFkB (complex), NA, malondialdehyde	CYP1A2, CYP2C9, CYP1A1, sulfaphenazole, furafylline	NADPH oxidase, ESR2, ESR1, estrogen receptor, PI3K (complex), TNF	(73, 74)
(k)	Naringenin	480–41-1	272.25	Tomentose Pummelo PeelFortune’s Drynaria Rhizome	↑TGF-β1/Smad pathway→EMT	Akt, TLR2, APOB, triacylglycerol, MTTP, PON1, SMAD3, PI3K (complex), TSLP, PPARGC1A, cholesterol ester, LDLR, mir-29, SOAT2, TLR4	K-252	Estrogen receptor, ESR2	(75–79)
(l)	Isoliquiritigenin	961–29-5	256.25	Liquorice Root	↑CD4+ ↑ CD8+ T ↑p38 MAPK ↓Autophagy	PTGS2, Vegf, MMP9, MMP2, reactive oxygen species, NOS2, KDR, BAX, SERPINF1, IL6, CCN2, IL1B, BCL2, hydrogen peroxide, IL5	–	Estrogen receptor, NMDA Receptor	(80–85)
(m)	Xanthohumol	6,754-58-1	354.4	Lightyellow Sophra Root	↓NF-κB、 ↓VEGF、IL-8、 ↓JAK/STAT、↓Notch1pathway	NFkB (complex), RUNX2, SREBF1, Akt, C18 acylcarnitine, C5 acylcarnitine, C17 acylcarnitine, linolenic acid hydroperoxide, C16:3 acylcarnitine, C8 acylcarnitine, C8:1 acylcarnitine, C20:4 dicarboxylic acid, C24 acylcarnitine, C15 acylcarnitine, C20:4 acylcarnitine	nitro-blue tetrazolium	Sec23, Sec24c	(86–91)
(n)	Silibinin	22,888–70-6	482.44	Blessed Thistle Fruit	↓Bcl-2 、 Bcl-xl ↑Bax 、 Bim ↓Autophagy	CDKN1A, CDKN1B, CDK4, CCND1, NOS2, CDK2, PTGS2, CCNB1, CASP3, IGFBP3, MMP9, CDK6, CCNE1, MYC, Vegf	–	–	(92–96)
(o)	Puerarin	3,681-99-0	416.38	Lobed Kudzuvine Root	↓Bcl-2/Bax、↓Akt/mTOR	CASP3, CASP9, NOS3, reactive oxygen species, ABCB1, BAX, TXN, BCL2, PTGS2, RETN, CYP19A1, Anti-inflammatory Cytokine, LEP, MAPK1, GSK3B	-	–	(97)
(p)	Icariin	489–32-7	676.66	Short-horned Epimedium Herb	↑ Bax Cleaved Caspase-3, ↓Bcl-2, ↑miR-9 ↓Wnt/β、NF-κB pathway	Alp, BCL2L1, ADCY2, ADCY9, ADCY10, COL1A2, RUNX2, Collagen Alpha1, Pka catalytic subunit, SLC2A4, Creb, cyclic AMP	–	–	(98–105)
(q)	Morusin	62,596–29-6	420.5	White Mulberry Root - bark	↓NF-κB、STAT-3	BCL2L1	–	–	(106)
(r)	Anthocyanins	14,051–53-7	287.2437	Flowering plant	Regulating Pancreatic Cancer Cell Migration	cholesterol, LEP, TRAF2, lipid, BDNF, DPP4	Magnesium, pyrogallol, carbon dioxide	–	(107)

#### 3.1.1 Apoptosis

Apoptosis, or programmed cell death, is a fundamental process disrupted in cancer cells, allowing them to evade normal cellular death mechanisms. Flavonoids significantly contribute to the regulation of apoptosis by targeting crucial pro- and anti-apoptotic molecules. Flavonoids such as EGCG, Apigenin, Baicalein and Quercetin are the natural compounds that inhibit BCL2 (a well-known anti-apoptotic protein) (106, 107), as reported in Wogonin-induced apoptosis of human hepatoma. These results permit apoptotic signals to take over and induce cell death in transformed cells by relieving the inhibitory effect of BCL2 on apoptosis (108, 109). On the other hand, flavonoids Silibinin and Icariin along with Rutin upregulate BAX expression further skewing balance towards apoptosis.

Also, activation of antiapoptotic protein BCL2L1 (*Bcl-xL*) is inhibited by EGCG, apigenin and quercetin helping the apoptotic pathways. Flavonoids activate *CASP3* (110, 111), a crucial death executor in caspase family. Icariin, Silibinin, Fisetin, Kaempferol and Morusin augment the activation of *CASP3* that promotes cell death with feature biochemical events such as cleavage protein cascades which allow key cellular proteins to become protease substrates making cancer cells disintegrate orderly.

#### 3.1.2 Inflammation

Cancer development and progression are accompanied by chronic inflammation. Flavonoids interact with key enzymes and cytokines in the upstream or downstream of inflammatory response pathways related to PC so could help alleviate an inflammation of PC. This includes Prostaglandin-Endoperoxide Synthase 2 (*PTGS2*), also known as *COX-2*, one of the main enzymes involved in pro-inflammatory prostaglandins production. Some of the flavonoids (Apigenin, Rutin, Myricetin) exhibited improved inhibition on *PTGS2* activity with some also promoting a decline in inflammation and hence lowered signals that promote cancer as well (112).

Furthermore, several bioflavonoids and flavanones have been found to induce downregulation of Interleukin-6 (*IL-6*) (22), a pleiotropic cytokine associated with immune response regulation as well as cancer-related inflammation, hence attenuating the maintenance of peritumoral pro-inflammatory milieu (which provides support for tumor growth) e.g., EGCG, Quercetin, Apigenin, Baicalein, and Wogonin.

In addition to inhibiting the activity of indoleamine 2,3-dioxygenase (IDO), an enzyme that facilitates immune evasion in tumors by metabolizing tryptophan into kynurenine, flavonoids also possess the ability to suppress tumor necrosis factor (TNF) (113), a pivotal pro-inflammatory cytokine involved in signaling pathways within normal human mesenchymal stem cells (NH-MSCs). By mitigating the actions of both IDO and TNF, these bioactive compounds may play a significant role in curtailing the inflammatory processes associated with breast cancer development.

Both Apigenin and Chrysin clearly suppress the expression of Intercellular Adhesion Molecule 1 (*ICAM1*), which regulates cell adhesion in many situations that promotes process such as immune cell recruitment for tumor microenvironment and also leads to Immune evasion by down regulating Nitric Oxide Synthase 2 (*NOS2*) downstream genes implicated with metastasis. Flavonoids such as EGCG, Baicalein, Wogonin, Isoliquiritigenin, and Silibinin assist in alleviating the inflammatory processes that promote cancer growth.

#### 3.1.3 Oxidative stress

Oxidative stress, characterized by an imbalance between the production of ROS and diminished antioxidant defenses, is closely linked to the initiation and progression of cancer. Flavonoids exert protective effects against oxidative stress by inhibiting specific target enzymes associated with their robust antioxidant activity. Flavonoids such as Baicalein, Wogonin, Isoliquiritigenin, and Silibinin have been shown to promote the expression of the anti-inflammatory and antioxidant enzyme Heme Oxygenase 1 (HMOX1) (114, 115). The upregulation of HMOX1 enhances the capacity of cells to scavenge ROS (116, 117), thereby mitigating oxidative damage—a critical factor in carcinogenesis. This modulation of oxidative stress by flavonoids underscores their potential role in cancer protection.

Furthermore, some flavonoids demonstrate significant binding affinities for estrogen receptors, specifically ESR1 (estrogen receptor *α*) and ESR2 (estrogen receptor *β*). Compounds such as Apigenin, Baicalein, Quercetin, and Naringenin have been shown to interact with these receptors, influencing cancer cell signaling and proliferation (118, 119). Also, Apigenin, Chrysin, Quercetin, and Rutin have been reported to interact with P-glycoprotein (Pgp), which is often overexpressed in drug-resistant cancer cell lines (120). These interactions suggest that flavonoids could function as chemo-sensitizing agents, potentially overcoming resistance to chemotherapy and improving treatment outcomes.

The role of flavonoids in cancer prevention and treatment is complex, as they exhibit both pro-oxidant and antioxidant properties depending on the cellular environment and concentration. While their antioxidant effects can protect cells from oxidative damage and reduce cancer risk, their pro-oxidant effects at higher concentrations may induce stress in cancer cells, potentially enhancing the efficacy of treatments. This dual nature underscores the potential of flavonoids as context-dependent agents, capable of both protecting healthy cells and sensitizing cancer cells to therapeutic interventions.

In this table, a range of different Flavonoids were selected to give an idea on overall and key characteristics including chemical identifiers (e.g., CAS number, molecular weight) as well as the source from which they can be found. This review lists the concrete directions on how these flavonoids modify key PC processes, apoptosis, inflammation, oxidative stress and other cancer-associated signaling pathways. The table also points out the molecular targets for flavonoids (*BCL2, BAX, PTGS2 and HMOX1*), along with their effect on signaling pathways such PI3K/AKT/mTOR or NF-κB and specific receptors or enzymes to which these polyphenols bind that demonstrate its implication in cellular regulation as well as potential applications in PC treatment.

### 3.2 Related pathways and effects of flavonoids against PC

This section discusses the most important signaling pathways controlled by flavonoids with PC treatment. These pathways are related to cancer cell proliferation, migration and autophagy. Research has suggested that flavonoids are capable of inhibiting these processes, leading to anti-tumor properties. A visual representation of the regulatory function of flavonoids in these pathways is included in Figure 2 where their regulation on the processes have been delineated.

#### 3.2.1 NF-κB pathway inhibition

The NF-κB pathway orchestrates the control of inflammation, cell survival and proliferation which are all critical elements involved in progression to PC (121). Flavonoids, including EGCG, Apigenin, Baicalein, Quercetin, Icariin, and Morusin, inhibit this pathway by preventing the phosphorylation of on *NF-κB* regulatory protein— *NF-κB* which sequesters and locks *NF-κB* in cytoplasm (122). These compounds have exerted their efficacy through the modulation of proinflammatory cytokines (*IL6, TNF*) and oncogenes (*Cyclin D1, MMP-9 and VEGF*) by inhibiting *NF-kB* activationas illustrated in Figure 2. This result in suppressed tumor growth, migration and survival. Thus, the inhibition *NF-κB* by flavones indicates an anti-inflammatory and antitumor environment in tumor microenvironment.

#### 3.2.2 PI3K/AKT pathway inhibition

The phosphoinositide 3-kinase (PI3K)/ AKT pathway is important for cell growth, proliferation and survival in most cancer types including pancreatic adenocarcinoma (123, 124). In the current study, 7 flavonoids (EGCG, Apigenin, Quercetin, Kaempferol, Fisetin, Puerarin, and Icariin) were identified to blunt this pathway by specifically inactivating *PI3K* and subsequently blocking *AKT* activation along with its downstream targets (*mTOR*, *Cyclin D1/D2*) as illustrated in Figure 2. This suppress of mTOR signaling pathway will result in a lower protein synthesis, cell proliferation, and an enlarged apoptosis rate within cancer cells (125, 126). Perhaps, the flavonoids suppress the proliferation and survival of cancer cells due to inhibition of PI3K/AKT signaling pathway that is associated with cell apoptosis and tumorigenesis as presented in PC model.

#### 3.2.3 SHH pathway inhibition

Sonic Hedgehog (SHH) Signaling Pathway, secreted in both paracrine and autocrine fashions, SHH is another significant player that contributes to cancer development, especially cell growth and tissue regeneration. This pathway could be blocked by flavonoids such as Quercetin and Baicalein, through the direct repression of *GLI1* transcription factor needed for cell proliferation and differentiation, targeting the *SMO* receptor that activation leads to *GLI1* family proteins (43). These flavonoids not only prevent PC tumorigenesis through reducing SHH pathway activation, impede the tumor growth and proliferation of PC cells as displayed in Figure 2.

#### 3.2.4 TGF-*β*/Smad pathway inhibition

TGF-β/Smad is one of the most important pathways that regulates cell growth and differentiation, whose dysregulation has been inextricably intertwined with cancer development. It was also demonstrated that Naringenin and Baicalein block this pathway by targeting the TGF-β receptor resulting in impaired downstream *Smad2/3* complex activation following treatment (127). It inhibits epithelial-mesenchymal transition (EMT) and consequent cell invasion (128), which results in metastasis suppression. Figure 2 indicates a mechanism of flavonoids to block TGF-β/Smad pathway for inhibition in cancer cell migration and invasion.

#### 3.2.5 p38-MAPK pathway inhibition

The p38-MAPK pathway is an important regulator of autophagy and apoptosis in cancer cells. These compounds, Fisetin, Apigenin, and Isoliquiritigenin also blocked the phosphorylation of *p38* signaling pathway to release apoptosis proteins *Caspase 3* and *Bax/Bcl2* (106). These in turn trigger autophagy and apoptosis, leading to a decreased ability of the cancer cell to survive. Figure 2 flavonoid regulation on cell survival and autophagy by p38-MAPK pathway.

#### 3.2.6 IL6/JAK/STAT3 pathway inhibition

IL6/JAK/STAT3 pathway plays a significant role in promoting cell survival, proliferation, and inflammation in PC. Quercetin inhibits this pathway by blocking the activation of *IL6R*, which prevents the subsequent phosphorylation of *JAK* and *STAT3*, key proteins responsible for activating oncogenic transcription factors (129). This inhibition results in decreased *STAT3* activation and reduced cancer cell survival, as shown in Figure 2. By targeting the IL6/JAK/STAT3 pathway, flavonoids reduce the pro-survival signaling that typically supports tumor growth and resistance to apoptosis.

#### 3.2.7 Notch pathway inhibition

Notch signaling pathway is involved in cell differentiation and survival, particularly in maintaining the self-renewal capacity of cancer stem cells. Flavonoids such as Icariin and Xanthohumol inhibit this pathway, impairing cancer cell survival and self-renewal by reducing the expression of *Notch1* and its downstream targets (130). The suppression of the Notch pathway limits the regenerative capabilities of cancer cells, making it a crucial target for flavonoid-based interventions, as demonstrated in Figure 2.

#### 3.2.8 P38-MAPK pathway inhibition

Fisetin, Apigenin and Isoliquiritigenin flavonoids induces the inactivation of one-member p38-MAPK pathway involved with cell proliferation and survival under stress. Flavonoids could induce autophagy and apoptosis in p38 signaling way, especially by regulating proteins such as *Beclin-1* (119), *ROS* (128), *Caspase-3* (131), and *Bax*. The suppression of this route results in diminished cancer cell viability and facilitates programmed cell death, hence underscoring the therapeutic potential of flavonoids in regulating cancer cell survival (refer to Figure 2).

Figure 2 depicts the influence of flavonoids, including EGCG, Quercetin, Apigenin, Baicalein, Kaempferol, and Puerarin, on critical signaling pathways that facilitate prostate cancer growth. The colorful boxes signify various flavonoid chemicals, while the arrows illustrate the direction of pathway regulation or inhibition.

## 4 Discussion

### 4.1 Clinical applications of flavonoids in PC

Flavonoids have shown considerable promise in preclinical and clinical studies for their potential role in managing PC. By targeting multiple signaling pathways, they exhibit synergistic effects when used in combination with conventional chemotherapies. Below is an overview of the clinical significance of key flavonoids, along with relevant clinical trial data supporting their therapeutic potential.

#### 4.1.1 Quercetin and rutin

Quercetin, a dietary flavonoid found in fruits and vegetables, has demonstrated substantial anti-cancer effects in both *in vitro* and *in vivo* studies. Its ability to modulate the NF-κB and PI3K/AKT pathways results in the inhibition of cancer cell proliferation and induction of apoptosis (53, 113). In clinical settings, a Phase I clinical trial (NCT01912820) evaluated the safety and efficacy of Quercetin in combination with gemcitabine for treating patients with advanced PC. This trial revealed that Quercetin enhanced the cytotoxic effects of gemcitabine, thereby improving therapeutic response and overall patient outcomes. Rutin, a glycoside of Quercetin, has also shown strong anti-inflammatory and anti-cancer properties, though its clinical application in PC remains under investigation (132).

#### 4.1.2 Baicalein and Wogonin

Baicalein derived from *Scutellaria baicalensis*, and their structurally similar flavonoid, Wogonin, target multiple pathways, *including NF-κB, PI3K/AKT*, and *MAPK* (133). These flavonoids are known for reducing tumor growth, suppressing metastasis, and enhancing apoptosis in PC cells. Early clinical studies have investigated Baicalein’s role in reducing chemotherapy-induced toxicity and improving patient tolerance to treatment. The potential of Wogonin is also being explored for its efficacy in overcoming chemoresistance, though clinical trials are still in the preliminary stages (134).

#### 4.1.3 Epigallocatechin gallate (EGCG)

EGCG, the principal catechin in g*reen tea*, has been shown to inhibit the PI3K/AKT/mTOR and NF-κB signaling pathways, thereby suppressing tumor cell proliferation and inducing apoptosis. While EGCG has been extensively studied in the context of other cancers, its specific application in PC treatment is still emerging. Preliminary studies suggest that EGCG supplementation may enhance chemotherapeutic response and reduce treatment-associated side effects (11), making it a candidate for further investigation.

#### 4.1.4 Apigenin, Chrysin, and Fisetin

Apigenin, Chrysin, and Fisetin are flavonoids that inhibit the NF-κB, STAT3, and PI3K/AKT pathways, leading to reduced cancer cell proliferation and enhanced apoptosis. Apigenin has been shown to sensitize PC cells to gemcitabine, making it a potential candidate for combination therapies (135–137). Although clinical trials focusing on these specific flavonoids in PC are limited, their well-documented preclinical efficacy supports the need for future clinical investigations to validate their therapeutic potential.

#### 4.1.5 Myricetin, Kaempferol, and Naringenin

Myricetin, Kaempferol, and Naringenin, found in various fruits and vegetables, have demonstrated potent anti-cancer effects by targeting the PI3K/AKT, MAPK, and TGF-*β* pathways (138). Kaempferol, in particular, has been studied for its ability to reduce cell proliferation and induce apoptosis, and early-phase clinical studies are evaluating its potential as a complementary therapy (139). Naringenin has shown the ability to enhance chemotherapeutic effects and reduce drug resistance, making it a candidate for future clinical trials focused on overcoming chemoresistance in PC.

#### 4.1.6 Silibinin, Xanthohumol, and Isoliquiritigenin

Silibinin, a major component of milk thistle, has been shown to inhibit PI3K/AKT, STAT3, and TGF-β pathways, reducing cancer cell proliferation and inducing apoptosis (140). Clinical trials have highlighted Silibinin’s potential in reducing chemotherapy toxicity and improving patient outcomes. Xanthohumol, found in hops, and Isoliquiritigenin, from licorice, modulate NF-κB and PI3K/AKT pathways, resulting in decreased tumor growth and metastasis (141, 142). Although clinical trials for these flavonoids in PC are limited, their strong preclinical profiles suggest potential for future investigations.

#### 4.1.7 Morusin and Anthocyanins

Morusin, a flavonoid isolated from *Morus alba* (mulberry tree), and Anthocyanins, a group of pigments found in berries and grapes, are strong inhibitors of the NF-κB and STAT3 pathways (143). By reducing cancer cell proliferation and suppressing metastasis, these flavonoids show promise as complementary agents in PC treatment. Their potent antioxidant properties further support their role in mitigating oxidative stress and inflammation in the tumor microenvironment. Clinical trials for Anthocyanins (NCT01692340) have shown their ability to reduce cancer biomarkers and enhance the overall health status of patients, highlighting their therapeutic potential (144).

#### 4.1.8 Puerarin and Icariin

Puerarin and Icariin, derived from traditional Chinese medicinal herbs, have shown the ability to modulate the MAPK and PI3K/AKT pathways (145, 146), leading to suppression of tumor growth and reduced cancer cell invasion. Clinical studies have indicated that Puerarin can protect normal cells while selectively targeting cancer cells, making it an attractive candidate for further development. Icariin has been shown to enhance the sensitivity of PC cells to conventional treatments, and ongoing clinical trials are evaluating its role in overcoming chemoresistance (147).

The clinical application of these flavonoids in PC is supported by both preclinical and emerging clinical evidence. However, challenges such as low bioavailability and rapid metabolism must be addressed through advanced drug delivery systems and combinatory strategies. Continued research and well-designed clinical trials are essential to fully establish their role in effective PC management.

### 4.2 Challenges and future directions

However, despite the promising preclinical and clinical data to support their potential use in PC management, several obstacles are faced regarding the direct application of flavonoids clinically (148). Flavonoids show limited bioavailability, rapid metabolism, and insufficient solubility; however, recently these limitations have been overcome by the advent of innovative drug delivery systems like nanoparticles, liposomes, polymeric micelles to improve therapeutic efficacy. In addition, there has been no confirmation with clinical studies on the effective combination of flavonoids for existing chemotherapies and influence that optimal dosing schedules may offer reduced side effects.

## 5 Conclusion and perspectives

PC develop through multiple molecular pathways and interplay, make the pathogenesis of PC extremely intricate. Flavonoids are a family of natural substances with different chemical structures and abilities to act on several molecular pathways. A feature that fits the multi-factorial nature of PC development, a property referred to as polypharmacology. Flavonoids might potentially disrupt the complex chain of interrelated processes leading to cancer by targeting essential factors for cancer cell survival, proliferation, angiogenesis, and metastasis.

*Scutellaria baicalensis*, a widely used clinical compound, contains various bioactive constituents such as Baicalin, Wogonin, Quercetin, Fisetin, Naringenin, Isoliquiritigenin, Xanthohumol, Puerarin, Icariin, and mulberry, in addition to traditional Chinese medicinal compounds such as ginkgo, continues, liquorice, and *Sophora flavescens* (149). Notably, *Sophora flavescens* exhibits significant multitarget effects in the treatment of PC (150). The classification and mechanisms of action of flavonoids in the treatment of PC were systematically investigated. These effects include antioxidant and anti-inflammatory properties, inhibition of cell proliferation and induction of apoptosis, suppression of angiogenesis and cancer cell metastasis, as well as immune modulation.

These natural products have anti-cancer properties. They can be treated not only individually but also in combination. They can not only be combined with chemotherapy drugs to increase the sensitivity of cells to chemotherapy drugs but also be combined with biological materials to enhance the anti-cancer properties of drugs. Additionally, combinations of many flavonoids or the addition of new materials offer additional ways to target cancer cells. Their different actions have a synergistic effect on treatment.

As an auxiliary treatment for cancer, flavonoid-based traditional Chinese medicine plays a crucial role at various stages of tumor development, including post-surgery, radiotherapy, and chemotherapy. This review aims to enhance the understanding of Chinese herbal medicine as an adjuvant therapy throughout the entire cancer treatment process, rather than being limited to the terminal stages. Additionally, it offers valuable insights for the development of more effective anti-cancer drugs and highlights the potential for these treatments to improve the long-term management of cancer, enabling patients to manage the disease as a chronic condition.

In addition, the diversity of flavonoids could provide tailored treatment options for PC, and for individual patients, or intake through diet or supplements, flavonoids could control the development of PC to a certain extent, perhaps allowing high-risk populations to benefit. Early intervention, combined with the concept of “prevention before disease occurs” in traditional Chinese medicine, is a unique feature.

Since PC is often diagnosed in its late stages, introducing a daily intake of flavonoids in high-risk individuals—such as those with obesity, diabetes, chronic pancreatitis, or unhealthy lifestyles—could be explored as a complementary approach within the “prevention before disease occurs” framework. However, more clinical evidence is required to establish its effectiveness in reducing the risk of PC.

However, comprehensive studies are critical to determine the efficacy, safety, appropriate dosage levels, and potential synergies with existing treatments. Tailoring treatment strategies for specific patients is an important aspect of personalized medicine. This requires effective collaboration among traditional medicine researchers, doctors and practitioners. Combining rigorous scientific methods with existing traditional methods has the potential to further advance the understanding and application of flavonoids in PC treatment. Applying the concept of “prevention before disease occurs” in traditional Chinese medicine is expected to unleash the potential of its anti-PC strategy.

In conclusion, while flavonoids hold significant promise for the treatment of PC, their therapeutic potential is complex and requires further investigation. Specifically, aspects such as the appropriate clinical application stages, optimal dosage, and collaborative strategies in treatment need to be thoroughly explored to maximize their effectiveness.

## References

[1] Amini M Azizmohammad Looha M Rahimi Pordanjani S Asadzadeh Aghdaei H Pourhoseingholi MA. Global long-term trends and spatial cluster analysis of pancreatic cancer incidence and mortality over a 30-year period using the global burden of disease study 2019 data. PLoS One. (2023) 18:e0288755. doi: 10.1371/journal.pone.0288755, PMID: 37471411 PMC10358895

[2] McCubrey JA Abrams SL Follo MY Manzoli L Ratti S Martelli AM. Effects of chloroquine and hydroxychloroquine on the sensitivity of pancreatic cancer cells to targeted therapies. Adv Biol Regul. (2023) 87:100917. doi: 10.1016/j.jbior.2022.100917, PMID: 36243652

[3] Schepis T de Lucia SS Pellegrino A del Gaudio A Maresca R Coppola G. State-of-the-art and upcoming innovations in pancreatic Cancer care: a step forward to precision medicine. Cancers. (2023) 15:3423. doi: 10.3390/cancers15133423, PMID: 37444534 PMC10341055

[4] de Luna FCF Ferreira WAS Casseb SMM de Oliveira EHC. Anticancer potential of flavonoids: an overview with an emphasis on Tangeretin. Pharmaceuticals. (2023) 16:1229. doi: 10.3390/ph16091229, PMID: 37765037 PMC10537037

[5] Jamal A. Embracing Nature’s therapeutic potential: herbal medicine. Int J Multidisciplinary Sciences and Arts. (2023) 2:117–26. doi: 10.47709/ijmdsa.v2i1.2620

[6] Bailly C. The subgroup of 2′-hydroxy-flavonoids: molecular diversity, mechanism of action, and anticancer properties. Bioorg Med Chem. (2021) 32:116001. doi: 10.1016/j.bmc.2021.116001 33444847

[7] Mazumder A Sharma A Azad MA. A comprehensive review of the pharmacological importance of dietary flavonoids as Hepatoprotective agents. Evid Based Complement Alternat Med. (2023) 2023:117. doi: 10.1155/2023/4139117 PMC10147524 37123086

[8] Tiwari P Mishra KP. Role of plant-derived flavonoids in Cancer treatment. Nutr Cancer. (2023) 75:430–49. doi: 10.1080/01635581.2022.2135744 36264133

[9] Tarkaa C Oyaniyi D Salaam R. Elucidating the molecular targets of *Curcuma longa* for breast Cancer treatment using network pharmacology, molecular docking and molecular dynamics simulation. Precis Med Res. (2023) 5:8. doi: 10.53388/PMR20230008

[10] Monteiro F Shetty SS. Natural antioxidants as inhibitors of pyruvate kinase M2 in Warburg phenotypes. J Herbal Med. (2023) 42:100750. doi: 10.1016/j.hermed.2023.100750

[11] Gu Q du Q Xia L Lu X Wan X Shao Y. Mechanistic insights into EGCG’s preventive effects on obesity-induced precocious puberty through multi-omics analyses. Food Funct. (2024) 15:11169–85. doi: 10.1039/D4FO03844D 39445911

[12] Sadowska-Krępa E Kłapcińska B Pokora I Domaszewski P Kempa K Podgórski T. Effects of six-week *Ginkgo biloba* supplementation on aerobic performance, blood pro/antioxidant balance, and serum brain-derived neurotrophic factor in physically active men. Nutrients. (2017) 9:803. doi: 10.3390/nu9080803, PMID: 28933745 PMC5579597

[13] Tiwari SC Husain N. Biological activities and role of flavonoids in human health–a. Indian J Sci Res. (2017) 12:193–6. Available at: https://www.ijsr.in/upload/43871798640.pdf [Epub ahead of print].

[14] Tiwari P Mishra KP. Flavonoids sensitize tumor cells to radiation: molecular mechanisms and relevance to cancer radiotherapy. Int J Radiat Biol. (2020) 96:360–9. doi: 10.1080/09553002.2020.1694193 31738629

[15] Gan R-Y Li HB Sui ZQ Corke H. Absorption, metabolism, anti-cancer effect and molecular targets of epigallocatechin gallate (EGCG): an updated review. Crit Rev Food Sci Nutr. (2018) 58:924–41. doi: 10.1080/10408398.2016.1231168, PMID: 27645804

[16] Wei R Wirkus J Yang Z Machuca J Esparza Y Mackenzie GG. EGCG sensitizes chemotherapeutic-induced cytotoxicity by targeting the ERK pathway in multiple cancer cell lines. Arch Biochem Biophys. (2020) 692:108546. doi: 10.1016/j.abb.2020.108546 32818507 PMC7494570

[17] Hu L Xu X Chen X Qiu S Li Q Zhang D. Epigallocatechin-3-Gallate decreases hypoxia-inducible Factor-1 in pancreatic Cancer cells. Am J Chin Med. (2023) 51:761–77. doi: 10.1142/S0192415X23500362 36867109

[18] Kciuk M Alam M Ali N Rashid S Głowacka P Sundaraj R. Epigallocatechin-3-Gallate therapeutic potential in Cancer: mechanism of action and clinical implications. Molecules. (2023) 28:5246. doi: 10.3390/molecules28135246, PMID: 37446908 PMC10343677

[19] Suhail M Rehan M Tarique M Tabrez S Husain A Zughaibi TA. Targeting a transcription factor NF-κB by green tea catechins using in silico and in vitro studies in pancreatic cancer. Front Nutr. (2023) 9:1078642. doi: 10.3389/fnut.2022.1078642, PMID: 36712528 PMC9874859

[20] Cunha L Coelho SC Pereira MC Coelho MAN. Nanocarriers based on gold nanoparticles for epigallocatechin gallate delivery in cancer cells. Pharmaceutics. (2022) 14:491. doi: 10.3390/pharmaceutics14030491, PMID: 35335868 PMC8951757

[21] Liu S Xu ZL Sun L Liu Y Li CC Li HM. Epigallocatechin-3-gallate induces apoptosis in human pancreatic cancer cells via PTEN. Mol Med Rep. (2016) 14:599–605. doi: 10.3892/mmr.2016.5277, PMID: 27176210

[22] Shankar S Marsh L Srivastava RK. EGCG inhibits growth of human pancreatic tumors orthotopically implanted in Balb C nude mice through modulation of FKHRL1/FOXO3a and neuropilin. Mol Cell Biochem. (2013) 372:83–94. doi: 10.1007/s11010-012-1448-y, PMID: 22971992 PMC3508371

[23] Ashrafizadeh M Bakhoda MR Bahmanpour Z Ilkhani K Zarrabi A Makvandi P. Apigenin as tumor suppressor in cancers: biotherapeutic activity, nanodelivery, and mechanisms with emphasis on pancreatic cancer. Front Chem. (2020) 8:829. doi: 10.3389/fchem.2020.00829, PMID: 33195038 PMC7593821

[24] Pandey P Khan F Seifeldin SA Alshaghdali K Siddiqui S Abdelwadoud ME. Targeting Wnt/β-catenin pathway by flavonoids: implication for Cancer therapeutics. Nutrients. (2023) 15:2088. doi: 10.3390/nu15092088, PMID: 37432240 PMC10181252

[25] Naso LG Ferrer EG Williams PA. Correlation of the anticancer and pro-oxidant behavior and the structure of flavonoid-oxidovanadium (IV) complexes. Coord Chem Rev. (2023) 492:215271. doi: 10.1016/j.ccr.2023.215271

[26] Tang J-Y Chuang YT Shiau JP Yen CY Chang FR Tsai YH. Connection between radiation-regulating functions of natural products and miRNAs targeting Radiomodulation and exosome biogenesis. Int J Mol Sci. (2023) 24:12449. doi: 10.3390/ijms241512449, PMID: 37569824 PMC10419287

[27] Feng Y-B Chen L Chen FX Yang Y Chen GH Zhou ZH. Immunopotentiation effects of apigenin on NK cell proliferation and killing pancreatic cancer cells. Int J Immunopathol Pharmacol. (2023) 37:03946320231161174. doi: 10.1177/03946320231161174, PMID: 36848930 PMC9974612

[28] Wu D-G Yu P Li JW Jiang P Sun J Wang HZ. Apigenin potentiates the growth inhibitory effects by IKK-β-mediated NF-κB activation in pancreatic cancer cells. Toxicol Lett. (2014) 224:157–64. doi: 10.1016/j.toxlet.2013.10.007, PMID: 24148603

[29] He J Ning C Wang Y Ma T Huang H Ge Y. Natural plant flavonoid apigenin directly disrupts Hsp90/Cdc37 complex and inhibits pancreatic cancer cell growth and migration. J Funct Foods. (2015) 18:10–21. doi: 10.1016/j.jff.2015.06.052

[30] Moslehi M Rezaei S Talebzadeh P Ansari MJ Jawad MA Jalil AT. Apigenin in cancer therapy: prevention of genomic instability and anticancer mechanisms. Clin Exp Pharmacol Physiol. (2023) 50:3–18. doi: 10.1111/1440-1681.13725, PMID: 36111951

[31] Iizumi Y Oishi M Taniguchi T Goi W Sowa Y Sakai T. The flavonoid apigenin downregulates CDK1 by directly targeting ribosomal protein S9. PLoS One. (2013) 8:e73219. doi: 10.1371/journal.pone.0073219, PMID: 24009741 PMC3756953

[32] Dewi C Fristiohady A Amalia R Khairul Ikram NK Ibrahim S Muchtaridi M. Signaling pathways and natural compounds in triple-negative breast cancer cell line. Molecules. (2022) 27:3661. doi: 10.3390/molecules27123661, PMID: 35744786 PMC9227697

[33] Gupta S Buttar HS Kaur G Tuli HS. Baicalein: promising therapeutic applications with special reference to published patents. Pharm Patent Anal. (2022) 11:23–32. doi: 10.4155/ppa-2021-0027, PMID: 35345898

[34] Sharifi-Rad J Herrera-Bravo J Salazar LA Shaheen S Abdulmajid Ayatollahi S Kobarfard F. The therapeutic potential of wogonin observed in preclinical studies. Evid Based Complement Alternat Med. (2021) 2021:1–9. doi: 10.1155/2021/9935451, PMID: 34221094 PMC8221866

[35] Yang JY Li M Zhang CL Liu D. Pharmacological properties of baicalin on liver diseases: a narrative review. Pharmacol Rep. (2021) 73:1230–9. doi: 10.1007/s43440-021-00227-1 33595821 PMC8460515

[36] Rais J Khan H Arshad M. The role of phytochemicals in Cancer prevention: a review with emphasis on Baicalein, Fisetin, and Biochanin A. Curr Top Med Chem. (2023) 23:1123–35. doi: 10.2174/1568026623666230516161827, PMID: 37194231

[37] Imran M Aslam Gondal T Atif M Shahbaz M Batool Qaisarani T Hanif Mughal M. Apigenin as an anticancer agent. Phytother Res. (2020) 34:1812–28. doi: 10.1002/ptr.6647 32059077

[38] Ma D Chen S Wang H Wei J Wu H Gao H. Baicalein induces apoptosis of pancreatic cancer cells by regulating the expression of miR-139-3p and miR-196b-5p. Front Oncol. (2021) 11:653061. doi: 10.3389/fonc.2021.653061, PMID: 33996574 PMC8120266

[39] Johnson JL De Mejia EG. Flavonoid apigenin modified gene expression associated with inflammation and cancer and induced apoptosis in human pancreatic cancer cells through inhibition of GSK-3β/NF-κ B signaling cascade. Mol Nutr Food Res. (2013) 57:2112–27. doi: 10.1002/mnfr.201300307, PMID: 23943362

[40] Madunić J Madunić IV Gajski G Popić J Garaj-Vrhovac V. Apigenin: a dietary flavonoid with diverse anticancer properties. Cancer Lett. (2018) 413:11–22. doi: 10.1016/j.canlet.2017.10.041, PMID: 29097249

[41] Pu WL Luo YY Bai RY Guo AW Zhou K Zhang YS. Baicalein inhibits acinar-to-ductal metaplasia of pancreatic acinal cell AR42J via improving the inflammatory microenvironment. J Cell Physiol. (2018) 233:5747–55. doi: 10.1002/jcp.26293, PMID: 29155449

[42] Zhou R-T He M Yu Z Liang Y Nie Y Tai S. Baicalein inhibits pancreatic cancer cell proliferation and invasion via suppression of NEDD9 expression and its downstream Akt and ERK signaling pathways. Oncotarget. (2017) 8:56351–63. doi: 10.18632/oncotarget.16912, PMID: 28915595 PMC5593566

[43] Song L Chen X Wang P Gao S Qu C Liu L. Effects of baicalein on pancreatic cancer stem cells via modulation of sonic hedgehog pathway. Acta Biochim Biophys Sin. (2018) 50:586–96. doi: 10.1093/abbs/gmy045, PMID: 29697746

[44] Liu H Dong Y Gao Y du Z Wang Y Cheng P. The fascinating effects of baicalein on cancer: a review. Int J Mol Sci. (2016) 17:1681. doi: 10.3390/ijms17101681, PMID: 27735841 PMC5085714

[45] Takahashi H Chen MC Pham H Angst E King JC Park J. Baicalein, a component of Scutellaria baicalensis, induces apoptosis by Mcl-1 down-regulation in human pancreatic cancer cells. Biochim Biophy Acta Mol Cell Res. (2011) 1813:1465–74. doi: 10.1016/j.bbamcr.2011.05.003 PMC3123440 21596068

[46] Liu X Peng X Cen S Yang C Ma Z Shi X. Wogonin induces ferroptosis in pancreatic cancer cells by inhibiting the Nrf2/GPX4 axis. Front Pharmacol. (2023) 14:1129662. doi: 10.3389/fphar.2023.1129662, PMID: 36909174 PMC9992170

[47] Banik K Khatoon E Harsha C Rana V Parama D Thakur KK. Wogonin and its analogs for the prevention and treatment of cancer: a systematic review. Phytother Res. (2022) 36:1854–83. doi: 10.1002/ptr.7386 35102626

[48] Zhang X Chen Y Li X Xu H Yang J Wang C. Carrier-free self-assembled nanomedicine based on celastrol and galactose for targeting therapy of hepatocellular carcinoma via inducing ferroptosis. Eur J Med Chem. (2024) 267:116183. doi: 10.1016/j.ejmech.2024.116183, PMID: 38354520

[49] Zhang T Liu M Liu Q Xiao GG. Wogonin increases gemcitabine sensitivity in pancreatic cancer by inhibiting Akt pathway. Front Pharmacol. (2022) 13:1068855. doi: 10.3389/fphar.2022.1068855, PMID: 36618921 PMC9816391

[50] Lim HK Kwon HJ Lee GS Moon JH Jung J. Chrysin-induced G protein-coupled estrogen receptor activation suppresses pancreatic Cancer. Int J Mol Sci. (2022) 23:9673. doi: 10.3390/ijms23179673, PMID: 36077069 PMC9456301

[51] Zhou L Yang C Zhong W Wang Q Zhang D Zhang J. Chrysin induces autophagy-dependent ferroptosis to increase chemosensitivity to gemcitabine by targeting CBR1 in pancreatic cancer cells. Biochem Pharmacol. (2021) 193:114813. doi: 10.1016/j.bcp.2021.114813 34673014

[52] Rolnik A Żuchowski J Stochmal A Olas B. Quercetin and kaempferol derivatives isolated from aerial parts of *Lens culinaris* Medik as modulators of blood platelet functions. Ind Crop Prod. (2020) 152:112536. doi: 10.1016/j.indcrop.2020.112536

[53] Samantaray A Pradhan D Nayak NR Chawla S Behera B Mohanty L. Nanoquercetin based nanoformulations for triple negative breast cancer therapy and its role in overcoming drug resistance. Discov Oncol. (2024) 15:452. doi: 10.1007/s12672-024-01239-y, PMID: 39287822 PMC11408462

[54] Asgharian P Tazehkand AP Soofiyani SR Hosseini K Martorell M Tarhriz V. Quercetin impact in pancreatic cancer: an overview on its therapeutic effects. Oxidative Med Cell Longev. (2021) 2021:266. doi: 10.1155/2021/4393266, PMID: 34777687 PMC8580629

[55] Guo Y Tong Y Zhu H Xiao Y Guo H Shang L. Quercetin suppresses pancreatic ductal adenocarcinoma progression via inhibition of SHH and TGF-β/Smad signaling pathways. Cell Biol Toxicol. (2021) 37:479–96. doi: 10.1007/s10565-020-09562-0, PMID: 33070227

[56] Lan C-Y Chen SY Kuo CW Lu CC Yen GC. Quercetin facilitates cell death and chemosensitivity through RAGE/PI3K/AKT/mTOR axis in human pancreatic cancer cells. J Food Drug Anal. (2019) 27:887–96. doi: 10.1016/j.jfda.2019.07.001, PMID: 31590760 PMC9306979

[57] Yu D Ye T Xiang Y Shi Z Zhang J Lou B. Quercetin inhibits epithelial–mesenchymal transition, decreases invasiveness and metastasis, and reverses IL-6 induced epithelial–mesenchymal transition, expression of MMP by inhibiting STAT3 signaling in pancreatic cancer cells. Onco Targets Ther. (2017) 10:4719–29. doi: 10.2147/OTT.S136840, PMID: 29026320 PMC5626388

[58] Nwaeburu CC Abukiwan A Zhao Z Herr I. Quercetin-induced miR-200b-3p regulates the mode of self-renewing divisions in pancreatic cancer. Mol Cancer. (2017) 16:1–10. doi: 10.1186/s12943-017-0589-8 28137273 PMC5282715

[59] Serri C Quagliariello V Iaffaioli RV Fusco S Botti G Mayol L. Combination therapy for the treatment of pancreatic cancer through hyaluronic acid-decorated nanoparticles loaded with quercetin and gemcitabine: a preliminary in vitro study. J Cell Physiol. (2019) 234:4959–69. doi: 10.1002/jcp.27297, PMID: 30334571

[60] Yi T Zhang W Hua Y Xin X Wu Z Li Y. Rutin alleviates lupus nephritis by inhibiting T cell oxidative stress through PPARγ. Chem Biol Interact. (2024) 394:110972. doi: 10.1016/j.cbi.2024.110972, PMID: 38555047

[61] Dogra A. Phytotherapeutic potential of Rutin against xenobiotic-induced toxicities in preclinical models. Food Rev Intl. (2024) 40:1–24. doi: 10.1080/87559129.2023.2279623

[62] Hassan H., Neuroprotective and anti-inflammatory potentials of rutin in an in vitro model of Alzheimer’s disease. (2024).

[63] Gatasheh MK. Identifying key genes against rutin on human colorectal cancer cells via ROS pathway by integrated bioinformatic analysis and experimental validation. Comput Biol Chem. (2024) 112:108178. doi: 10.1016/j.compbiolchem.2024.108178 39191167

[64] Huo M Xia A Cheng W Zhou M Wang J Shi T. Rutin promotes pancreatic cancer cell apoptosis by upregulating miRNA-877-3p expression. Molecules. (2022) 27:2293. doi: 10.3390/molecules27072293, PMID: 35408691 PMC9000526

[65] Huang C Zhou S Zhang C Jin Y Xu G Zhou L. ZC3H13-mediated N6-methyladenosine modification of PHF10 is impaired by fisetin which inhibits the DNA damage response in pancreatic cancer. Cancer Lett. (2022) 530:16–28. doi: 10.1016/j.canlet.2022.01.013, PMID: 35033590

[66] Xiao Y Liu Y Gao Z Li X Weng M Shi C. Fisetin inhibits the proliferation, migration and invasion of pancreatic cancer by targeting PI3K/AKT/mTOR signaling. Aging. (2021) 13:24753–67. doi: 10.18632/aging.203713, PMID: 34821587 PMC8660603

[67] Ding G Xu X Li D Chen Y Wang W Ping D. Fisetin inhibits proliferation of pancreatic adenocarcinoma by inducing DNA damage via RFXAP/KDM4A-dependent histone H3K36 demethylation. Cell Death Dis. (2020) 11:893. doi: 10.1038/s41419-020-03019-2, PMID: 33093461 PMC7582166

[68] Jia S Xu X Zhou S Chen Y Ding G Cao L. Fisetin induces autophagy in pancreatic cancer cells via endoplasmic reticulum stress-and mitochondrial stress-dependent pathways. Cell Death Dis. (2019) 10:142. doi: 10.1038/s41419-019-1366-y, PMID: 30760707 PMC6374379

[69] Murtaza I Adhami VM Hafeez BB Saleem M Mukhtar H. Fisetin, a natural flavonoid, targets chemoresistant human pancreatic cancer AsPC-1 cells through DR3-mediated inhibition of NF-κB. Int J Cancer. (2009) 125:2465–73. doi: 10.1002/ijc.24628, PMID: 19670328 PMC2944651

[70] Phillips P Sangwan V Borja-Cacho D Dudeja V Vickers SM Saluja AK. Myricetin induces pancreatic cancer cell death via the induction of apoptosis and inhibition of the phosphatidylinositol 3-kinase (PI3K) signaling pathway. Cancer Lett. (2011) 308:181–8. doi: 10.1016/j.canlet.2011.05.002, PMID: 21676539 PMC3126884

[71] Zhang Z Guo Y Chen M Chen F Liu B Shen C. Kaempferol potentiates the sensitivity of pancreatic cancer cells to erlotinib via inhibition of the PI3K/AKT signaling pathway and epidermal growth factor receptor. Inflammopharmacology. (2021) 29:1587–601. doi: 10.1007/s10787-021-00848-1 34322786

[72] Wang F Wang L Qu C Chen L Geng Y Cheng C. Kaempferol induces ROS-dependent apoptosis in pancreatic cancer cells via TGM2-mediated Akt/mTOR signaling. BMC Cancer. (2021) 21:1–11. doi: 10.1186/s12885-021-08158-z 33845796 PMC8042867

[73] Bhia M Motallebi M Abadi B Zarepour A Pereira-Silva M Saremnejad F. Naringenin nano-delivery systems and their therapeutic applications. Pharmaceutics. (2021) 13:291. doi: 10.3390/pharmaceutics13020291, PMID: 33672366 PMC7926828

[74] Arafah A Rehman MU Mir TM Wali AF Ali R Qamar W. Multi-therapeutic potential of naringenin (4′, 5, 7-trihydroxyflavonone): experimental evidence and mechanisms. Plan Theory. (2020) 9:1784. doi: 10.3390/plants9121784, PMID: 33339267 PMC7766900

[75] Lee J Kim D-H Kim JH. Combined administration of naringenin and hesperetin with optimal ratio maximizes the anti-cancer effect in human pancreatic cancer via down regulation of FAK and p38 signaling pathway. Phytomedicine. (2019) 58:152762. doi: 10.1016/j.phymed.2018.11.022, PMID: 31005717

[76] Ren Q Chen G Wan Q Xiao L Zhang Z Feng Y. Unravelling the role of natural and synthetic products as DNA topoisomerase inhibitors in hepatocellular carcinoma. Bioorg Chem. (2024) 153:107860. doi: 10.1016/j.bioorg.2024.107860, PMID: 39442463

[77] Ren Q Li M Deng Y Lu A Lu J. Triptolide delivery: nanotechnology-based carrier systems to enhance efficacy and limit toxicity. Pharmacol Res. (2021) 165:105377. doi: 10.1016/j.phrs.2020.105377, PMID: 33484817

[78] Wang L Yang R Yuan B Liu Y Liu C. The antiviral and antimicrobial activities of licorice, a widely-used Chinese herb. Acta Pharm Sin B. (2015) 5:310–5. doi: 10.1016/j.apsb.2015.05.005 26579460 PMC4629407

[79] Dinnen RD Mao Y Fine RL. The use of fluorescent probes in the study of reactive oxygen species in pancreatic cancer cells. Methods Protoc. (2013) 980:321–9. doi: 10.1007/978-1-62703-287-2_18 23359163

[80] Giuliani CM Dass CR. Autophagy and cancer: taking the ‘toxic’out of cytotoxics. J Pharm Pharmacol. (2013) 65:777–89. doi: 10.1111/jphp.12034 23647671

[81] Zhang Z Yung KK-L Ko JK-S. Therapeutic intervention in cancer by isoliquiritigenin from licorice: a natural antioxidant and redox regulator. Antioxidants. (2022) 11:1349. doi: 10.3390/antiox11071349, PMID: 35883840 PMC9311861

[82] Peng F du Q Peng C Wang N Tang H Xie X. A review: the pharmacology of isoliquiritigenin. Phytother Res. (2015) 29:969–77. doi: 10.1002/ptr.5348 25907962

[83] Zhang Z Chen WQ Zhang SQ Bai JX Liu B Yung KKL. Isoliquiritigenin inhibits pancreatic cancer progression through blockade of p38 MAPK-regulated autophagy. Phytomedicine. (2022) 106:154406. doi: 10.1016/j.phymed.2022.154406, PMID: 36029643

[84] Krajka-Kuźniak V Paluszczak J Baer-Dubowska W. Xanthohumol induces phase II enzymes via Nrf2 in human hepatocytes in vitro. Toxicol Vitro. (2013) 27:149–56. doi: 10.1016/j.tiv.2012.10.008, PMID: 23085367

[85] Kunnimalaiyaan S Trevino J Tsai S Gamblin TC Kunnimalaiyaan M. Xanthohumol-mediated suppression of Notch1 signaling is associated with antitumor activity in human pancreatic cancer cells. Mol Cancer Ther. (2015) 14:1395–403. doi: 10.1158/1535-7163.MCT-14-0915, PMID: 25887885 PMC4554525

[86] Cykowiak M Kleszcz R Kucińska M Paluszczak J Szaefer H Plewiński A. Attenuation of pancreatic cancer in vitro and in vivo via modulation of Nrf2 and NF-κB signaling pathways by natural compounds. Cells. (2021) 10:3556. doi: 10.3390/cells10123556, PMID: 34944062 PMC8700195

[87] Saito K Matsuo Y Imafuji H Okubo T Maeda Y Sato T. Xanthohumol inhibits angiogenesis by suppressing nuclear factor-κB activation in pancreatic cancer. Cancer Sci. (2018) 109:132–40. doi: 10.1111/cas.13441, PMID: 29121426 PMC5765302

[88] Li S Xu HX Wu CT Wang WQ Jin W Gao HL. Angiogenesis in pancreatic cancer: current research status and clinical implications. Angiogenesis. (2019) 22:15–36. doi: 10.1007/s10456-018-9645-2, PMID: 30168025

[89] Jiang W Zhao S Xu L Lu Y Lu Z Chen C. The inhibitory effects of xanthohumol, a prenylated chalcone derived from hops, on cell growth and tumorigenesis in human pancreatic cancer. Biomed Pharmacother. (2015) 73:40–7. doi: 10.1016/j.biopha.2015.05.020, PMID: 26211581

[90] Nambiar D Prajapati V Agarwal R Singh RP. *In vitro* and *in vivo* anticancer efficacy of silibinin against human pancreatic cancer BxPC-3 and PANC-1 cells. Cancer Lett. (2013) 334:109–17. doi: 10.1016/j.canlet.2012.09.004, PMID: 23022268

[91] Lee Y Chun HJ Lee KM Jung YS Lee J. Silibinin suppresses astroglial activation in a mouse model of acute Parkinson’s disease by modulating the ERK and JNK signaling pathways. Brain Res. (2015) 1627:233–42. doi: 10.1016/j.brainres.2015.09.029 26434409

[92] Bai Y Chen J Hu W Wang L Wu Y Yu S’. Silibinin therapy improves *Cholangiocarcinoma* outcomes by regulating ERK/mitochondrial pathway. Front Pharmacol. (2022) 13:847905. doi: 10.3389/fphar.2022.847905, PMID: 35401195 PMC8983842

[93] Ray PP Islam MA Islam MS Han A Geng P Aziz MA. A comprehensive evaluation of the therapeutic potential of silibinin: a ray of hope in cancer treatment. Front Pharmacol. (2024) 15:1349745. doi: 10.3389/fphar.2024.1349745 38487172 PMC10937417

[94] Feng W Cai D Zhang B Lou G Zou X. Combination of HDAC inhibitor TSA and silibinin induces cell cycle arrest and apoptosis by targeting survivin and cyclinB1/Cdk1 in pancreatic cancer cells. Biomed Pharmacother. (2015) 74:257–64. doi: 10.1016/j.biopha.2015.08.017 26349994

[95] Zhu H Xiao Y Guo H Guo Y Huang Y Shan Y. The isoflavone puerarin exerts anti-tumor activity in pancreatic ductal adenocarcinoma by suppressing mTOR-mediated glucose metabolism. Aging. (2021) 13:25089–105. doi: 10.18632/aging.203725, PMID: 34863080 PMC8714170

[96] He C Wang Z Shi J. Pharmacological effects of icariin. Adv Pharmacol. (2020) 87:179–203. doi: 10.1016/bs.apha.2019.10.004 32089233

[97] Fan C Yang Y Liu Y Jiang S di S Hu W. Icariin displays anticancer activity against human esophageal cancer cells via regulating endoplasmic reticulum stress-mediated apoptotic signaling. Sci Rep. (2016) 6:21145. doi: 10.1038/srep21145, PMID: 26892033 PMC4759694

[98] Ke Z Liu J Xu P Gao A Wang L Ji L. The Cardioprotective effect of icariin on ischemia–reperfusion injury in isolated rat heart: potential involvement of thePI3K‐Akt signaling pathway. Cardiovasc Ther. (2015) 33:134–40. doi: 10.1111/1755-5922.12121, PMID: 25847837

[99] Wang G-Q Li DD Huang C Lu DS Zhang C Zhou SY. Icariin reduces dopaminergic neuronal loss and microglia-mediated inflammation *in vivo* and *in vitro*. Front Mol Neurosci. (2018) 10:441. doi: 10.3389/fnmol.2017.00441, PMID: 29375304 PMC5767257

[100] Zheng X Li D Li J Wang B Zhang L Yuan X. Optimization of the process for purifying icariin from Herba Epimedii by macroporous resin and the regulatory role of icariin in the tumor immune microenvironment. Biomed Pharmacother. (2019) 118:109275. doi: 10.1016/j.biopha.2019.109275 31382128

[101] Omura N Li CP Li A Hong SM Walter K Jimeno A. Genome-wide profiling at methylated promoters in pancreatic adenocarcinoma. Cancer Biol Ther. (2008) 7:1146–56. doi: 10.4161/cbt.7.7.6208, PMID: 18535405 PMC2763640

[102] Amidfar M Karami Z Kheirabadi GR Afshar H Esmaeili A. Expression of Bcl-2 and Bax genes in peripheral blood lymphocytes of depressed and nondepressed individuals. J Res Med Sci. (2019) 24:41. doi: 10.4103/jrms.JRMS_811_17 31160908 PMC6540770

[103] Huang Q Huchen YC Dai LM Dai LP. Effect of icariin on proliferation and apoptosis of pancreatic cancer cells BxPC-3 is related to upregulation of miR-9. Chin J Immunol. (2021) 37:289–94. doi: 10.3969/j.issn.1000-484X.2021.03.006

[104] Agarwal S Mohamed MS Mizuki T Maekawa T Sakthi Kumar D. Chlorotoxin modified morusin–PLGA nanoparticles for targeted glioblastoma therapy. J Mater Chem B. (2019) 7:5896–919. doi: 10.1039/C9TB01131E 31423502

[105] Mostafa H Behrendt I Meroño T González-Domínguez R Fasshauer M Rudloff S. Plasma anthocyanins and their metabolites reduce in vitro migration of pancreatic cancer cells, PANC-1, in a FAK-and NF-kB dependent manner: results from the ATTACH-study a randomized, controlled, crossover trial in healthy subjects. Biomed Pharmacother. (2023) 158:114076. doi: 10.1016/j.biopha.2022.114076, PMID: 36516693

[106] Dong M Yang Z Gao Q Deng Q Li L Chen H. Protective effects of Isoliquiritigenin and Licochalcone B on the Immunotoxicity of BDE-47: antioxidant effects based on the activation of the Nrf2 pathway and inhibition of the NF-kappaB pathway. Antioxidants. (2024) 13:445. doi: 10.3390/antiox13040445 38671893 PMC11047486

[107] Afroze N Sundaram MK Raina R Jathan J Bhagavatula D Haque S. Concurrent treatment of flavonol with chemotherapeutics potentiates or counteracts the therapeutic implications in cervical cancer cells. Minerva Biotecnol. (2023) 35:2938. doi: 10.23736/S2724-542X.22.02938-8

[108] Han S-H Lee JH Woo JS Jung GH Jung SH Han EJ. Myricetin induces apoptosis through the MAPK pathway and regulates JNK-mediated autophagy in SK-BR-3 cells. Int J Mol Med. (2022) 49:1–11. doi: 10.3892/ijmm.2022.5110 35234274 PMC8904074

[109] Alyami BA Zaki M Youns M Amin B Abdou R Dawoud M. Rutin inhibits hepatic and pancreatic Cancer cell proliferation by inhibiting CYP3A4 and GST. Ind J Pharm Edu Res. (2023) 57:s411–8. doi: 10.5530/ijper.57.2s.48

[110] Wei J Yu W Hao R Fan J Gao J. Anthocyanins from *Aronia melanocarpa* induce apoptosis in Caco-2 cells through Wnt/β-catenin signaling pathway. Chem Biodivers. (2020) 17:e2000654. doi: 10.1002/cbdv.202000654, PMID: 33016000

[111] Zhang X Liu J Zhang P Dai L Wu Z Wang L. Silibinin induces G1 arrest, apoptosis and JNK/SAPK upregulation in SW1990 human pancreatic cancer cells. Oncol Lett. (2018) 15:9868–76. doi: 10.3892/ol.2018.8541, PMID: 29805688 PMC5958732

[112] Li N Chen S Deng W Gong Z Guo Y Zeng S. Kaempferol attenuates gouty arthritis by regulating the balance of Th17/Treg cells and secretion of IL-17. Inflammation. (2023) 46:1–16. doi: 10.1007/s10753-023-01849-8 37311931

[113] Chen J Jiang M Ying Y Ji Y Chi Y Tao L. Network pharmacological mechanism analysis and evidence-based medical validation of Dahuang Mudan decoction in the treatment of acute pancreatitis. Medicine. (2024) 103:e39679. doi: 10.1097/MD.0000000000039679, PMID: 39287237 PMC11404899

[114] Lu L Li Y Dong Q Fang J Chen A Lan Z. Wogonin inhibits oxidative stress and vascular calcification via modulation of heme oxygenase-1. Eur J Pharmacol. (2023) 958:176070. doi: 10.1016/j.ejphar.2023.176070, PMID: 37739306

[115] Wu S Lu H Bai Y. Nrf2 in cancers: a double-edged sword. Cancer Med. (2019) 8:2252–67. doi: 10.1002/cam4.2101, PMID: 30929309 PMC6536957

[116] Rai P. Role of heat shock proteins in oncogenesis and strategy for treating cancers using *Drosophila* model. Proc Indian Natl Sci Acad. (2023) 89:247–53. doi: 10.1007/s43538-023-00166-w

[117] Ashrafizadeh M Zhang W Zou R Sethi G Klionsky DJ Zhang X. A bioinformatics analysis, pre-clinical and clinical conception of autophagy in pancreatic cancer: complexity and simplicity in crosstalk. Pharmacol Res. (2023) 194:106822. doi: 10.1016/j.phrs.2023.106822 37336429

[118] Yang L Chen L Chen T Gao X Xiong Y. The crosstalk between classic cell signaling pathways, non-coding RNAs and ferroptosis in drug resistance of tumors. Cell Signal. (2022) 102:110538. doi: 10.1016/j.cellsig.2022.110538 36436800

[119] Devis-Jauregui L Eritja N Davis ML Matias-Guiu X Llobet-Navàs D. Autophagy in the physiological endometrium and cancer. Autophagy. (2021) 17:1077–95. doi: 10.1080/15548627.2020.1752548, PMID: 32401642 PMC8143243

[120] Li Q Liao S Pang D Li E Liu T Liu F. The transported active mulberry leaf phenolics inhibited adipogenesis through PPAR-gamma and leptin signaling pathway. J Food Biochem. (2022) 46:e14270. doi: 10.1111/jfbc.14270, PMID: 35702955

[121] Pu W-L Bai RY Zhou K Peng YF Zhang MY Hottiger MO. Baicalein attenuates pancreatic inflammatory injury through regulating MAPK, STAT 3 and NF-κB activation. Int Immunopharmacol. (2019) 72:204–10. doi: 10.1016/j.intimp.2019.04.018, PMID: 30999210

[122] Uwagawa T Yanaga K. Effect of NF-κB inhibition on chemoresistance in biliary–pancreatic cancer. Surg Today. (2015) 45:1481–8. doi: 10.1007/s00595-015-1129-z, PMID: 25673034

[123] Gao A-M Ke ZP Shi F Sun GC Chen H. Chrysin enhances sensitivity of BEL-7402/ADM cells to doxorubicin by suppressing PI3K/Akt/Nrf2 and ERK/Nrf2 pathway. Chem Biol Interact. (2013) 206:100–8. doi: 10.1016/j.cbi.2013.08.008, PMID: 23994249

[124] LiuCCaoYZuoYZhangCRenSZhangX. Hybridization-based discovery of novel quinazoline-2-indolinone derivatives as potent and selective PI3Kα inhibitors. J Adv Res. (2024). doi: 10.1016/j.jare.2024.03.00238471647

[125] Liu S Wang XJ Liu Y Cui YF. PI3K/AKT/mTOR signaling is involved in (−) epigallocatechin-3-gallate-induced apoptosis of human pancreatic carcinoma cells. Am J Chin Med. (2013) 41:629–42. doi: 10.1142/S0192415X13500444, PMID: 23711146

[126] Chen Y Yang J Wang C Wang T Zeng Y Li X. Aptamer-functionalized triptolide with release controllability as a promising targeted therapy against triple-negative breast cancer. J Exp Clin Cancer Res. (2024) 43:207. doi: 10.1186/s13046-024-03133-5, PMID: 39054545 PMC11270970

[127] Li ZQ Yu B Cai ZY Wang YB Zhang X Zhou B. Naringenin inhibits thoracic aortic aneurysm formation in mice with Marfan syndrome. Beijing Da Xue Xue Bao. (2022) 54:896–906. doi: 10.19723/j.issn.1671-167X.2022.05.017 PMID: 36241232 PMC9568379

[128] Qiu M Ma K Zhang J Zhao Z Wang S Wang Q. Isoliquiritigenin as a modulator of the Nrf2 signaling pathway: potential therapeutic implications. Front Pharmacol. (2024) 15:1395735. doi: 10.3389/fphar.2024.1395735, PMID: 39444605 PMC11496173

[129] Xiong F Shen K Long D Zhou S Ruan P Xin Y. Quercetin ameliorates lupus symptoms by promoting the apoptosis of senescent Tfh cells via the Bcl-2 pathway. Immun Ageing. (2024) 21:69. doi: 10.1186/s12979-024-00474-9, PMID: 39407236 PMC11476537

[130] Cai J Qiao Y Chen L Lu Y Zheng D. Regulation of the notch signaling pathway by natural products for cancer therapy. J Nutr Biochem. (2024) 123:109483. doi: 10.1016/j.jnutbio.2023.109483, PMID: 37848105

[131] Alzahrani S Said E Ajwah SM Alsharif SY el-Bayoumi KS Zaitone SA. Isoliquiritigenin attenuates inflammation and modulates Nrf2/caspase-3 signalling in STZ-induced aortic injury. J Pharm Pharmacol. (2021) 73:193–205. doi: 10.1093/jpp/rgaa056, PMID: 33793806

[132] Weiz G González AL Mansilla IS Fernandez-Zapico ME Molejón MI Breccia JD. Rutinosides-derived from Sarocladium strictum 6-O-alpha-rhamnosyl-beta-glucosidase show enhanced anti-tumoral activity in pancreatic cancer cells. Microb Cell Factories. (2024) 23:133. doi: 10.1186/s12934-024-02395-0, PMID: 38720294 PMC11077868

[133] Ma S Wei T Zhang B Zhang Y Lai J Qu J. Integrated pharmacokinetic properties and tissue distribution of multiple active constituents in Qing-Yi recipe: a comparison between granules and decoction. Phytomedicine. (2024) 129:155645. doi: 10.1016/j.phymed.2024.155645, PMID: 38643714

[134] Yang H Liu C Lin X Li X Zeng S Gong Z. Wogonin inhibits the migration and invasion of fibroblast-like synoviocytes by targeting PI3K/AKT/NF-κB pathway in rheumatoid arthritis. Arch Biochem Biophys. (2024) 755:109965. doi: 10.1016/j.abb.2024.109965, PMID: 38552763

[135] Dunjic M Turini S Nejkovic L Sulovic N Cvetkovic S Dunjic M. Comparative molecular docking of *Apigenin* and *Luteolin* versus conventional ligands for TP-53, pRb, APOBEC3H, and HPV-16 E6: potential clinical applications in preventing gynecological malignancies. Curr Issues Mol Biol. (2024) 46:11136–55. doi: 10.3390/cimb46100661, PMID: 39451541 PMC11505693

[136] Sood A Mehrotra A Sharma U Aggarwal D Singh T Shahwan M. Advancements and recent explorations of anti-cancer activity of chrysin: from molecular targets to therapeutic perspective. Explor Target Antitumor Ther. (2024) 5:477–94. doi: 10.37349/etat.2024.00230, PMID: 38966181 PMC11220305

[137] Tavenier J Nehlin JO Houlind MB Rasmussen LJ Tchkonia T Kirkland JL. Fisetin as a senotherapeutic agent: evidence and perspectives for age-related diseases. Mech Ageing Dev. (2024) 222:111995. doi: 10.1016/j.mad.2024.111995, PMID: 39384074

[138] el-Gendy ZA Ammar NM Kassem AM Attia MS Afifi SM Ibrahim AH. Myricetin-loaded SBA-15 silica nanoparticles for enhanced management of pyrexia, pain, and inflammation through modulation of MAPK/NF-kappaB and COX-2/PGE-2 pathways: evidence from the biochemical, histological, and metabolomic analysis. Int J Pharm. (2024) 666:124775. doi: 10.1016/j.ijpharm.2024.124775, PMID: 39353498

[139] Zhao L Luo T Zhang H Fan X Zhang Q Chen H. Kaempferol enhances intestinal repair and inhibits the hyperproliferation of aging intestinal stem cells in *Drosophila*. Front Cell Dev Biol. (2024) 12:1491740. doi: 10.3389/fcell.2024.1491740, PMID: 39450272 PMC11499188

[140] Selc M Macova R Babelova A. Novel strategies enhancing bioavailability and Therapeutical potential of Silibinin for treatment of liver disorders. Drug Des Devel Ther. (2024) 18:4629–59. doi: 10.2147/DDDT.S483140, PMID: 39444787 PMC11498047

[141] He YF Liu YP Liao JZ Gan Y Li X Wang RR. Xanthohumol promotes Skp2 ubiquitination leading to the inhibition of glycolysis and tumorigenesis in ovarian Cancer. Am J Chin Med. (2024) 52:865–84. doi: 10.1142/S0192415X24500356 38790085

[142] Gong X Cai J Zheng W Huang J Chen T Chen W. Isoliquiritigenin alleviates SLC7A11-mediated efferocytosis inhibition to promote wounds healing in diabetes. Biomed Pharmacother. (2024) 180:117578. doi: 10.1016/j.biopha.2024.117578, PMID: 39427549

[143] Li D Yuan X Ma J Lu T Zhang J Liu H. Morusin, a novel inhibitor of ACLY, induces mitochondrial apoptosis in hepatocellular carcinoma cells through ROS-mediated mitophagy. Biomed Pharmacother. (2024) 180:117510. doi: 10.1016/j.biopha.2024.117510 39341077

[144] Moran NE Novotny JA Cichon MJ Riedl KM Rogers RB Grainger EM. Absorption and distribution kinetics of the 13C-labeled tomato carotenoid phytoene in healthy adults. J Nutr. (2016) 146:368–76. doi: 10.3945/jn.115.220525 26674763 PMC4725433

[145] Wang Q Shen ZN Zhang SJ Sun Y Zheng FJ Li YH. Protective effects and mechanism of puerarin targeting PI3K/Akt signal pathway on neurological diseases. Front Pharmacol. (2022) 13:1022053. doi: 10.3389/fphar.2022.1022053, PMID: 36353499 PMC9637631

[146] Jia X Li L Wang T Ma X Li C Liu M. Puerarin inhibits macrophage M1 polarization by combining STAT1 to reduce myocardial damage in EAM model mice. Biochem Biophys Res Commun. (2024) 733:150702. doi: 10.1016/j.bbrc.2024.150702, PMID: 39298917

[147] Wang Z Wang D Yang D Zhen W Zhang J Peng S. The effect of icariin on bone metabolism and its potential clinical application. Osteoporos Int. (2018) 29:535–44. doi: 10.1007/s00198-017-4255-1, PMID: 29110063

[148] Alhadrami HA Sayed AM Hassan HM Alhadrami AH Rateb ME. Molecular insights and inhibitory dynamics of flavonoids in targeting Pim-1 kinase for cancer therapy. Front Pharmacol. (2024) 15:1440958. doi: 10.3389/fphar.2024.1440958, PMID: 39434908 PMC11491346

[149] Qiao L Liu K Ren Y Liu Y Xu Z Wang S. *Scutellaria baicalensis* ameliorates allergic airway inflammation through agonism and transcriptional regulation of TAS2Rs. J Ethnopharmacol. (2024) 337:118881. doi: 10.1016/j.jep.2024.118881 39362328

[150] Cao X He Q. Anti-tumor activities of bioactive phytochemicals in *Sophora flavescens* for breast Cancer. Cancer Manag Res. (2020) 12:1457–67. doi: 10.2147/CMAR.S243127, PMID: 32161498 PMC7051174

